# DNA elements for constitutive androstane receptor- and pregnane X receptor-mediated regulation of bovine *CYP3A28* gene

**DOI:** 10.1371/journal.pone.0214338

**Published:** 2019-03-25

**Authors:** Mery Giantin, Jenni Küblbeck, Vanessa Zancanella, Viktoria Prantner, Fabiana Sansonetti, Axel Schoeniger, Roberta Tolosi, Giorgia Guerra, Silvia Da Ros, Mauro Dacasto, Paavo Honkakoski

**Affiliations:** 1 Department of Comparative Biomedicine and Food Science, University of Padua, Legnaro, Padua, Italy; 2 Faculty of Health Sciences, School of Pharmacy, University of Eastern Finland, Kuopio, Finland; 3 Institute of Biochemistry, University of Leipzig, Leipzig, Germany; Wayne State University, UNITED STATES

## Abstract

The regulation of cytochrome P450 3A (CYP3A) enzymes is established in humans, but molecular mechanisms of its basal and xenobiotic-mediated regulation in cattle are still unknown. Here, ~10 kbp of the bovine *CYP3A28* gene promoter were cloned and sequenced, and putative transcription factor binding sites were predicted. The *CYP3A28* proximal promoter (PP; -284/+71 bp) contained DNA elements conserved among species. Co-transfection of bovine nuclear receptors (NRs) pregnane X and constitutive androstane receptor (bPXR and bCAR) with various *CYP3A28* promoter constructs into hepatoma cell lines identified two main regions, the PP and the distal fragment F3 (-6899/-4937 bp), that were responsive to bPXR (both) and bCAR (F3 fragment only). Site-directed mutagenesis and deletion of NR motif ER6, hepatocyte nuclear factor 1 (HNF-1) and HNF-4 binding sites in the PP suggested either the involvement of ER6 element in bPXR-mediated activation or the cooperation between bPXR and liver-enriched transcription factors (LETFs) in PP transactivation. A putative DR5 element within the F3 fragment was involved in bCAR-mediated PP+F3 transactivation. Although DNA enrichment by anti-human NR antibodies was quite low, ChIP investigations in control and RU486-treated BFH12 cells, suggested that retinoid X receptor α (RXRα) bound to ER6 and DR5 motifs and its recruitment was enhanced by RU486 treatment. The DR5 element seemed to be recognized mainly by bCAR, while no clear-cut results were obtained for bPXR. Present results point to species-differences in CYP3A regulation and the complexity of bovine *CYP3A28* regulatory elements, but further confirmatory studies are needed.

## Introduction

The human cytochrome P450 3A (CYP3A) subfamily contains four isoforms (CYP3A4, CYP3A5, CYP3A7 and CYP3A43), and their expression and activity is influenced by genetic and non-genetic factors. CYP3A4 is one of the most important CYP isoform in the liver, where it plays a major role in the oxidative metabolism of 30–40% of all clinically used drugs as well as of important endogenous steroids. Multiple liver enriched transcription factors (LETFs) contribute to the complex regulation of human *CYP3A* genes: the xenosensors pregnane X-receptor (PXR, NR1I2), the constitutive androstane receptor (CAR, NR1I3), the hepatocyte nuclear factor-4 (HNF-4), HNF-3, the vitamin D receptor (VDR, NR1I1), and the CCAAT/enhancer binding protein-α (C/EBPα) [1–4].

CYP3A regulation has been extensively studied in humans and rodents; however, mechanistic studies in veterinary species are scant. Some papers about the regulation of pig CYP3As have been published, on account of its reliability as a preclinical animal model species [5,6]. Specifically, the transactivation profiles of porcine CAR and PXR were compared with human orthologs by using prototypical human ligands, and the abovementioned pig nuclear receptors (NRs) were proved similar to human counterparts in terms of ligand specificity [7–9]. Moreover, PXR has been demonstrated to play an important role in cytokine-mediated regulation of porcine *CYP3A29* [10–12]; finally, PXR and other LETFs like HNF-1α and Specificity protein 1 have been shown to contribute to basal and rifampicin (RIF)-mediated regulation of pig *CYP3A46* [13]. Overall, these data would be indicative of a similarity in the transcriptional regulation of porcine *CYP3A* and human *CYP3A4* [10].

Cattle is one of the most important food-producing species, and the bovine *CYP3A* locus contains at least four genes. Three of them (*CYP3A28*, *CYP3A38* and *CYP3A48*) have been identified and quantified in the liver [14,15]; the fourth one (*CYP3A24*) is still considered a predicted sequence. Understanding bovine *CYP3A* regulation is interesting, because CYP3A induction is dramatically species-specific. For example, exposure to the human CYP3A inducer dexamethasone (DEX) did not induce CYP3A in calves [16,17]. The human or mouse CYP3A inducers DEX, RIF and pregnenolone 16α-carbonitrile (PCN) failed to activate bovine PXR (bPXR), and only 4-[2,2-bis-(diethoxyphosphoryl)-ethenyl]-2,6-ditert-butylphenol (SR12813) and mifepristone (RU486) were proved as bPXR agonists [9]. However, the pleiotropic CYP inducer PB increases hepatic CYP3A mRNA and apoprotein levels [14]. In cattle husbandry, CYP3A enzymes are particularly involved in the metabolism of the macrocyclic lactone moxidectin (an endectocide) [18], tiamulin and macrolide antibiotics [19], and the ionophore monensin [20]. Moreover, they metabolize natural toxins like aflatoxin B1 [21] and ergot alkaloids [22]. In addition, age, gender and breed can influence bovine CYP3A expression and activity [23–26].

To overcome the lack of information on induction of bovine CYP3A, we report here sequencing, in silico analysis and characterization of ~10 kbp bovine *CYP3A28* promoter (orthologous of human *CYP3A4* [27]). Two clusters of NR and LETF binding sites were identified, and their roles in both basal and bPXR/bCAR-mediated gene activation were examined. Induction and chromatin immunoprecipitation (ChIP) studies, made on the bovine fetal hepatocyte-derived cell line BFH12 [28], would suggest the participation of bPXR and bCAR to *CYP3A28* regulation.

## Materials and methods

### Reagents

The established activators of human and mouse CAR and PXR have been reviewed [29–31]. DEX, PCN, RU486, SR12813 and their solvent dimethyl sulfoxide (DMSO) were obtained from Sigma-Aldrich (St. Louis, MO); RIF was purchased from Sanofi Aventis (Milan, Italy); 6-(4-chlorophenyl)imidazo[2,1-*b*][1,3]thiazole-5-carbaldehyde O-(3,4-dichlorobenzyl)oxime (CITCO) and 5-(3,4-dimethoxybenzyl)-3-phenyl-4,5-dihydroisoxazole (FL81) have been documented [32–34]. Oligonucleotides used in the study were synthesized by Integrated DNA Technologies (Leuven, Belgium). All other chemicals used in the study are commercially available and of molecular biology grade.

### Re-sequencing of the *Bos taurus CYP3A28* gene promoter

The cloning of ~10 kbp *CYP3A28* promoter is described in detail in S1 File. Briefly, liver DNA was isolated, and specific overlapping fragments amplified with proof-reading Phusion High-Fidelity DNA Polymerase (Thermo Fisher Scientific, Waltham, Massachusetts, USA) and using primers (S1 Table) designed on the available Hereford genome (GenBank Assembly ID: GCA_000003055.3). Each fragment was sequenced at least three times, and the final contig was submitted to GenBank (accession number KU696412).

### *CYP3A28* promoter sequence analysis

Putative binding sites for CAR and PXR heterodimers with RXR (DR3-5 and ER6-9 motifs) were identified with NUBIscan and NHR scan algorithms [35,36]. MatInspector 8.0.5 [37] was used to locate retinoid X receptor α (RXRα; NR2B1), HNF-3, HNF-4 and C/EBP binding sites. In each case, the search algorithms were optimized as detailed in S1 File.

### Reporter vectors and NR expression vectors

Selected DNA fragments, collectively covering the entire *CYP3A28* promoter and containing the putative NR and transcription factor (TF) binding sites, were amplified using TaKaRa LA Taq Hot Start DNA polymerase (TaKaRa Biotechnology Co., Otsu, Japan) and specific primers (S2 and S3 Tables). The fragments were purified and sequenced as before and subcloned 5’ of the *CYP3A28* proximal promoter (see S1 File for details).

The enhancer-driven luciferase reporters for full-length CAR (PBREM-tk-luc) and full-length PXR (XREM-3A4-luc) have been previously described [38,39] and used as positive controls for NR activation. The full-length cDNAs for *bPXR* and *bCAR* were inserted into CMV promoter-driven expression vectors [9].

### Generation of mutated TF sites and deletion constructs

In brief, site-directed mutagenesis of the ER6 and HNF-4 (DR1) sites was performed using mutagenic primers (S4 Table), amplification of DNA using Pfu DNA polymerase (Thermo Fisher Scientific) and elimination of template DNA with DpnI restriction endonuclease (Thermo Fisher Scientific). Deletions were done using inverse PCR method [40] using 5’-phosphorylated primers (S5 Table) that allow self-ligation of blunt-end PCR products. Competent E. coli DH5a cells were transformed with the generated constructs, plasmid DNAs were isolated and screened for correct mutations by restriction digestion and DNA sequencing. For details, see S1 File.

### Reporter gene assays in HepG2 and C3A cell lines

HepG2 (ATCC HB-8065) and C3A (ATCC CRL-10741) cells, characterized by very low constitutive *hCAR* and *hPXR* expression [39,41], were grown as reported by [41]. Before transfection, the cells were seeded onto 48-well plates (0.16 x 106 cells/cm^2^) and cultured overnight to have 50–70% confluence. HepG2 cells were transfected using PEI25 and a reverse transfection method [9], with control reporter pCMVβ (600 ng/well; Clontech Inc., Palo Alto, CA), *CYP3A28* reporter constructs, or with positive control reporters hPBREM-tk-luc or hCYP3A4-XREM-luc, or with negative control reporter pGL4.10-luc (500 ng/well) and expression vector pCI-neo, bCAR or bPXR (100 ng/well). C3A cells were transfected using the calcium phosphate precipitation method [42] with control reporter pCMVβ (150 ng/well) and the same *CYP3A28* reporter constructs or positive/negative control reporters (50 ng/well) and expression vectors (25 ng/well) as with HepG2. DMEM complemented with HyClone 5% delipidated serum (GE Healthcare) was used for the treatment with the PXR agonist SR12813 (10 μM) or its vehicle (DMSO, 0.1%). After 24 hours, cells were lysed and assayed for luciferase and β-galactosidase activities with the VICTOR2 multiplate reader (Perkin Elmer Wallac, Turku, Finland) as described before [42].

### Induction studies in BFH12 cell line

The novel bovine SV40 large T-antigen-transduced fetal hepatocyte-derived cell line BFH12 [28] was used to evaluate the capability of established human and mouse PXR (DEX, PCN, RIF, RU486 and SR12813) and CAR (CITCO and FL81) activators to induce bovine *CYP3A28* expression. BFH12 cells (passages 16–21) were cultured in Williams’ E medium containing 5% heat-inactivated fetal bovine serum (FBS), 1% penicillin/streptomycin, 2 mM L-alanyl-L-glutamine, 100 nM DEX and 0.2 U/mL insulin, at 37°C and 5% CO_2_ in a humidified atmosphere as described [28]. Cells were seeded onto 6-well plates (5 x 10^4^ cells/well). At day 4, the medium was changed with complete medium but without DEX. At day 7, stock solutions of PXR ligands, solubilized in DMSO and diluted in culture medium, were added into the wells. Cells treated with 0.1% DMSO (final concentration) were used as control.

Preliminary induction studies were performed using DEX, PCN, RIF, RU486 and SR12813 at a fixed concentration (10 μM), according to [9], and different time points (0, 1, 3, 6, 12 and 24 hours). Subsequently, to optimize ChIP conditions, a time point of 6 hours was chosen and different concentrations of NR ligands were tested. For RIF and RU486, 1, 2.5, 5, 10, 25, 50 and 100 μM were selected, while 1, 2.5, 5, 10 and 25 μM were chosen for SR12813. Concerning bCAR ligands, two time points (6 and 12 hours) and the concentrations 0.03, 0.1, 0.3 and 1 μM CITCO and 1, 3, 10 and 30 μM FL81 were used [9]. At least three independent experiments were performed. At the end of the incubation, the medium was removed, cells were washed with PBS and scraped off, re-suspended in RLT buffer (Qiagen, Hilden, Germany) containing 2-mercaptoethanol and stored at -20°C until use.

Total RNA was extracted with the RNeasy Mini kit (Qiagen) following manufacturer’s instructions and quantified by NanoDrop 1000 Spectrophotometer (Thermo Fisher Scientific). Complementary DNA (1 μg) was synthetized using the High Capacity cDNA Reverse Transcription kit (Applied Biosystems, Foster City, CA). The quantitative real-time PCR (qPCR) amplification was carried out in a final volume of 10 μL, using 12.5 ng of cDNA, the Power SYBR Green PCR Master Mix (Applied Biosystems) and Stratagene Mx3000P thermal cycler (Agilent Technologies, Santa Clara, California, United States). Standard qPCR conditions were used. For the amplification of *RXRα*, the gene-specific primers forward (F): 5’-GCGTACTGCAAACACAAGTACC- 3’ and reverse (R): 5’-AGGCACTTGAGGCCAATG-3’ were selected, while for *CYP2B22*, *CYP3A28*, *CAR*, *PXR* and *RPLP0* (internal control gene), previously published bovine primers were used [15,17,25]. Different concentrations of F and R primers were used: 50 nM/50 nM for *CAR* assay, 300 nM/300 nM for *CYP2B22*, *CYP3A28*, *RXRα* and *RPLP0*, 600 nM/600 nM for PXR. For each qPCR assay, negative controls (with either total RNA or water as template) were run. PCR amplification was performed in duplicate. The ΔΔCt method [43] was used to analyze gene expression results.

### Chromatin immunoprecipitation (ChIP) assay

BFH12 cells were seeded in 75 cm^2^ flasks at a density of 5 x 10^3^ cells/cm^2^. At day 4, the medium was replaced with fresh medium without DEX. At day 7, cells were incubated for 6 hours with 100 μM RU486, 30 μM FL81 or 0.1% DMSO. Then, the cells, after trypsinization, were subjected to ChIP assay. The process for ChIP assay is detailed in S1 File. Briefly, chromatin was cross-linked with 1% formaldehyde and cross-linking was quenched by addition of 125 mM glycine. Nuclei were then isolated and chromatin was sheared by sonication and subjected to immunoprecipitation using anti-hCAR, anti-hPXR and anti-hRXR antibodies. Anti-histone H3 and beads in absence of any antibody (no-antibody control) were used as positive and negative ChIP controls, respectively. Precipitated DNA fragments were then amplified in qPCR with specific primers flanking ER6 and DR5 elements in the proximal and F3 fragments, respectively (S6 Table).

### Data analysis

All transfections, apart from the initial screening of *CYP3A28* fragment activity in HepG2 cells, were performed at least in triplicates. Data were normalized to the pGL4.10 empty vector and pCI-neo when no bPXR or bCAR were co-transfected (basal activity); pGL4.10 co-transfected with bPXR and SR12183 activities were used for experiments involving bPXR; pGL4.10 co-transfected with bCAR were used to normalize luciferase activity in bCAR transfections. An arbitrary value of 100 was set for pGL4.10 + pCI-neo, pGL4.10 + bPXR + SR12183, pGL4.10 + bCAR.

For the statistical analysis of data from gene reporter assays and induction studies, the Student’s t-test or the analysis of variance (ANOVA), followed by the Tukey’s post-test was used (GraphPad Prism 4.02, San Diego, California). A *P* value < 0.05 was considered as statistically significant.

## Results

### Prediction of putative PXR, CAR and LETF binding sites

The re-sequencing successfully solved the two gaps present in the latest UMD3.1.1 genome release, upstream of the *CYP3A28* transcription start site (S1 Fig): the first N stretch starting at –1492 was a sequencing artefact (S2 Fig, panel A), while the second N stretch at base –2988 was substituted by a new genomic sequence (S2 Fig, panel B). Additionally, the Piedmontese contig contained five single nucleotide polymorphisms when compared to the Hereford sequence (S2 Fig, panel B), none of which affected the binding sites identified below.

**Fig 1:**
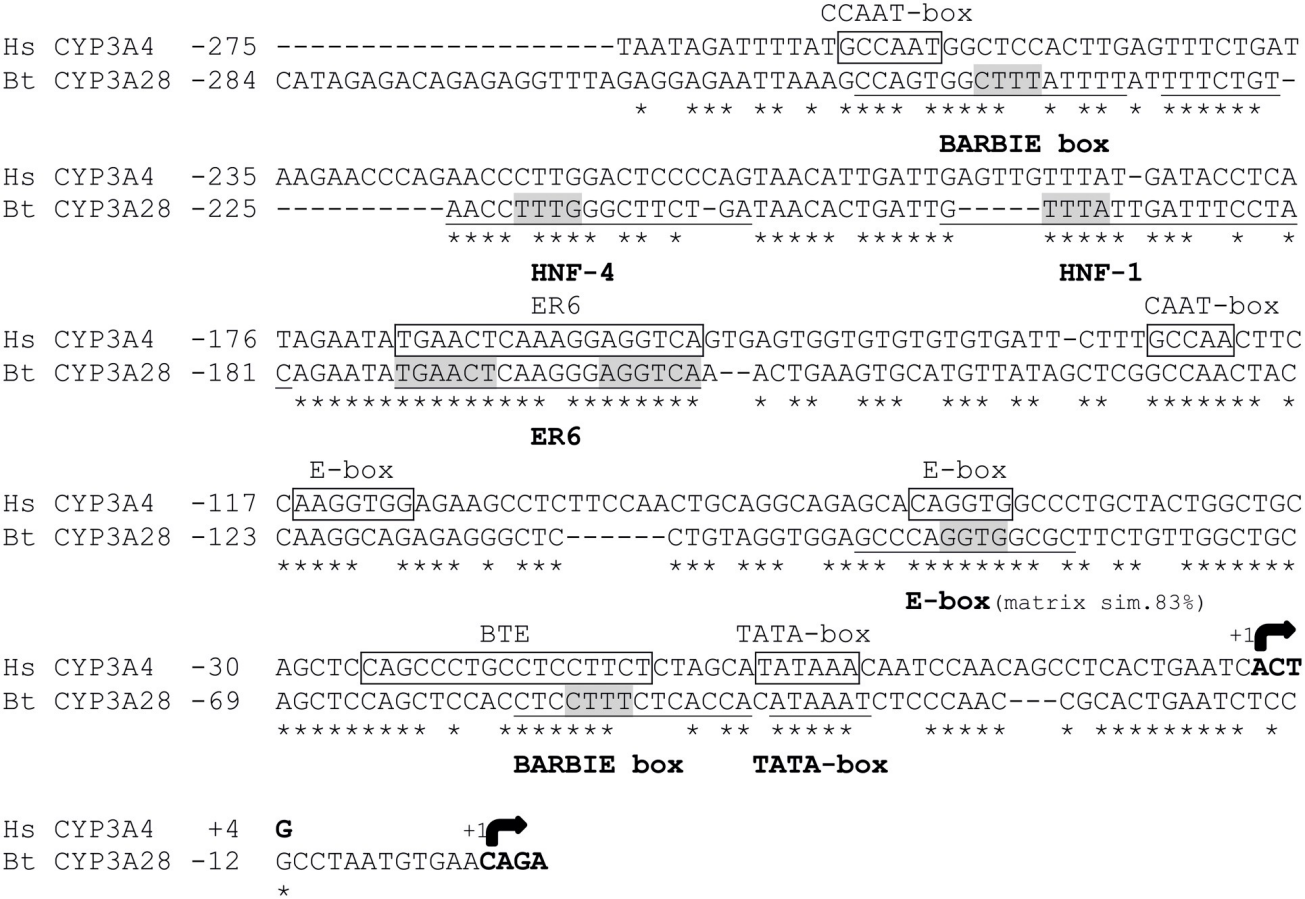
Sequence comparison and distribution of regulatory elements in the human *CYP3A4* and bovine *CYP3A28* proximal promoters. Identical nucleotides are denoted by asterisks. The transcription start sites are indicated by arrows. The sequence is numbered relative to the transcription start site taken as +1. The binding sites previously characterized in human proximal promoter are boxed (modified from [45]), the newly identified bovine elements are bolded and underlined. For the elements identified through MatInspector analysis, the nucleotides matching the matrix core are highlighted in grey colour.

**Fig 2:**
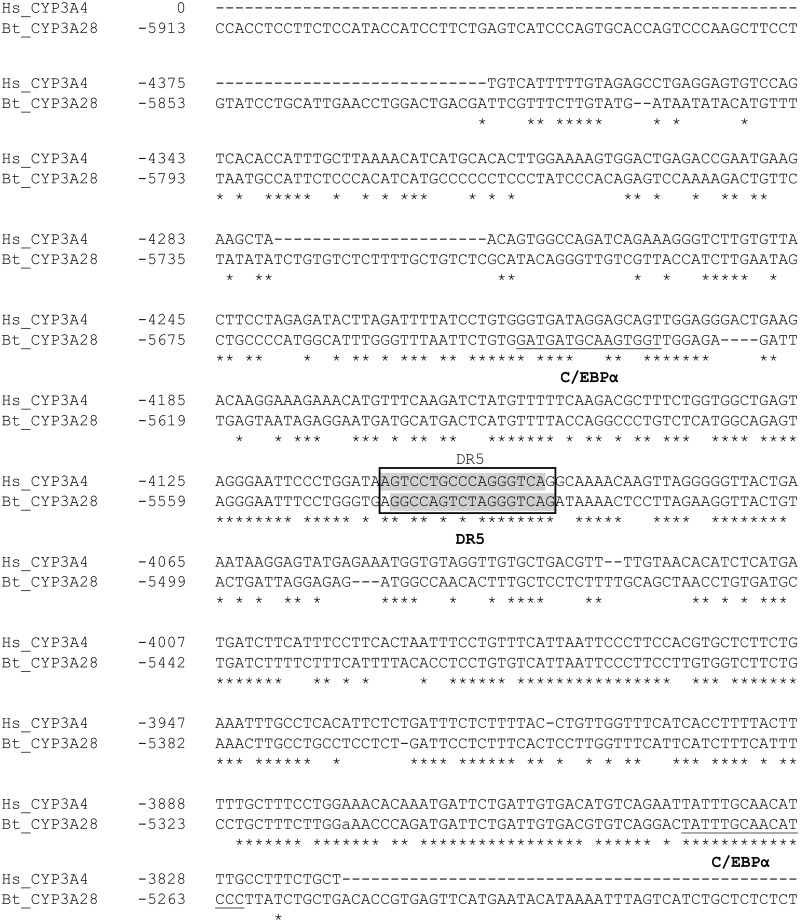
Sequence comparison and distribution of distal regulatory elements in the bovine *CYP3A28* F3 fragment and human *CYP3A4* promoter region. Identical nucleotides are denoted by asterisks. The sequence is numbered relative to the transcription start site taken as +1. The newly identified bovine elements (C/EBPα) are bolded and underlined. The DR5 element, identified in both species through NUBIscan analysis, is highlighted in grey colour and boxed.

The *in silico* analysis of ~10 kbp of the 5’ flanking sequence revealed response elements for PXR and CAR, and several putative binding sites for LETFs such as HNF-1, HNF-3, HNF-4 and C/EBPα (Table 1). The bovine *CYP3A28* PP contained an ER6-type motif for PXR and CAR at -173/-156 bp, which aligned well with the known human *CYP3A4* proER6 element (Fig 1). With ~74% overall identity, also the TATA-box (-40/-35 bp) and a proximal E-box (-90/-87 bp) were conserved between the human and bovine PPs. In contrast, the basic transcription element (BTE), a second E-box and a C/EBPα site present on human *CYP3A* genes [44,45], and the more distal XREM and CLEM modules in human *CYP3A4* gene were apparently not present in the bovine *CYP3A28* promoter. In fragment F2 (-4998/-3133 bp), one potential binding site for CAR at position -4338/-4323 bp was detected by MatInspector. This element was flanked by an HNF-3β site (5’) and two HNF-4 sites (3’). A putative binding site for C/EBPα was also predicted in F2 at -3288/-3273 bp. NUBIscan identified a DR5 motif at -5541/-5525 bp within the fragment F3 (-6899/-4937 bp) (Fig 2). Additionally, this region contained many LETF sites identified by MatInspector and Match softwares: the DR5 motif was flanked by two C/EBPα sites (5’) and a cluster of three HNF-3 and one HNF-4 element (3’) (Table 1 and Fig 2).

**Table 1:** Results from *in silico* screening of bovine *CYP3A28* gene promoter.

RE- and TF-name	Position in the Promoter Fragments						
	PP, lnPP	lnPP, F1	F1	F2	F3	F4	F5
CAR/RXRα ^(c)^	**/**	-876/-851	/	-4348/-4323	/	/	-9301/-9277
CAR/RXRα & HNF-4 ^(c)^	**/**	/	/	/	/	/	-9522/-9498
C/EBPα ^(c)^	**/**	/	/	-3288/-3273	-5275/-5260	-7485/-7471	/
					-5645/-5631		
DR-4 ^(a, b, c)^	**/**	-1250/-1234	/	/	/	/	/
DR-5 ^(a)^	**/**	/	/	/	-5541/-5525	/	/
ER-6 ^(a, b, c)^	-173/-156	/	/	/	/	/	/
HNF-1 ^(d)^	-196/-179	/	/	/	/	/	/
HNF-3 ^(c)^	/	/	/	/	-6091/-6075	/	-9313/-9298
							-10190/-10174
HNF-3β ^(c)^	/	/	-1362/-1345	-4986/-4969	-6007/-5991	-7022/-7006	-10006/-9990
			-3003/-2986		-6540/-6524		
HNF-4 ^(c, d)^	-225/-208 ^(c, d)^	-1143/-1118 ^(c)^	/	-3548/-3523 ^(c)^	-6114/-6090 ^(d)^	-8022/-7998 ^(c)^	/
HNF-4α ^(c)^	/	/	/	-4048/-4023	/	/	/
HNF-4 & DR-4 ^(a, b, c)^	/	/	/	/	/	/	-10248/-10244

The web-based software NUBIscan^a^ and

NHR-scan^b^ were used to identify PXR-RXR and CAR-RXR heterodimers DNA core binding motifs ER6-9 and DR3-5;

MatInspector^c^ was queried for RXR, HNF-3, HNF-4 and C/EBPα binding sites, while

Match^d^ through liver specific matrices identified HNF-1, HNF-3 and NHF-4 DNA binding elements.

### Screening of PXR- and CAR-responsive elements in *CYP3A28* promoter

**Fig 3:**
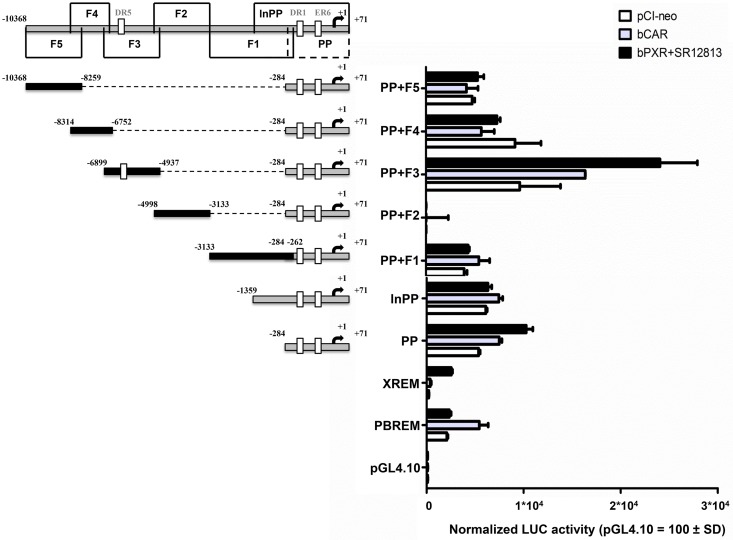
Screening for bPXR- and bCAR-responsive regions in *CYP3A28* promoter. A series of *CYP3A28* luciferase reporter gene constructs (PP, lnPP, PP+F1, PP+F2, PP+F3, PP+F4, PP+F5) was prepared as described in S1 File. Numbers indicate the positions relative to the transcriptional start site. HepG2 cells were transfected with the control reporter pCMVβ (600 ng/well), each reporter plasmids or PBREM-tk-luc (PBREM) and CYP3A4-XREM-luc (XREM) or with negative control reporter pGL4.10-luc (500 ng/well) and either bCAR and bPXR expression plasmids or pCI-neo empty vector (100 ng/well). After transfection, cells were treated with vehicle (0.1% DMSO) or SR12813 (10 μM) for 24 hours, and reporter activities were measured. Firefly luciferase activities were normalized with β-galactosidase activities. Data are expressed as relative activities to those in pGL4.10 transfected cells (= 100) for each condition (pCI-neo, bCAR or bPXR co-transfection). Data are the mean ± SD (n = 3 or 4) and representative of one assay.

The relevance of these putative NR sites was examined by reporter gene assays using the *CYP3A28* PP (-283/+71 bp) fused upstream of the luciferase reporter and appended by different upstream sequences (F1, F2, F3, F4, F5; Fig 3). This preliminary screening was carried out once in HepG2 cells (Fig 3). To ensure activation of bPXR, SR12813 was added into the culture medium, while bCAR is constitutively active [9]. The empty pGL4.10 reporter was not activated by bPXR or bCAR. The positive control plasmid PBREM-tk-luc was preferentially activated by bCAR, and the XREM-3A4-distal-luc activity was enhanced by bPXR as expected [32]. These results indicate the validity of our screening system. The *CYP3A28* PP was activated about 2- to 3-fold by bPXR, indicating the presence of PXR-responsive elements, while bCAR increased the reporter activity up to 40% when compared to empty pCI-neo. The addition of upstream elements (F1, -3133/-262 bp; F2, -4998/-3133 bp; F4, -8314/-6752 bp; F5, -10368/-8259 bp) to the PP appeared to decrease NR-dependent activation. This result was particularly evident for the fragment F2 (-4998/-3133 bp), where the basal activity and the NR-mediated activation of *CYP3A28* PP were completely abolished. This suggests the presence of silencing elements in F2. In contrast, bCAR and bPXR increased PP+F3 reporter activity of 70% and 150%, respectively, compared to empty pCI-neo. The responsiveness of F3 (-6899/-4937 bp) to both bPXR and bCAR indicated that the NR elements within this fragment should be analyzed further.

### Role of bPXR in the trans-activation of *CYP3A28* promoter

**Fig 4:**
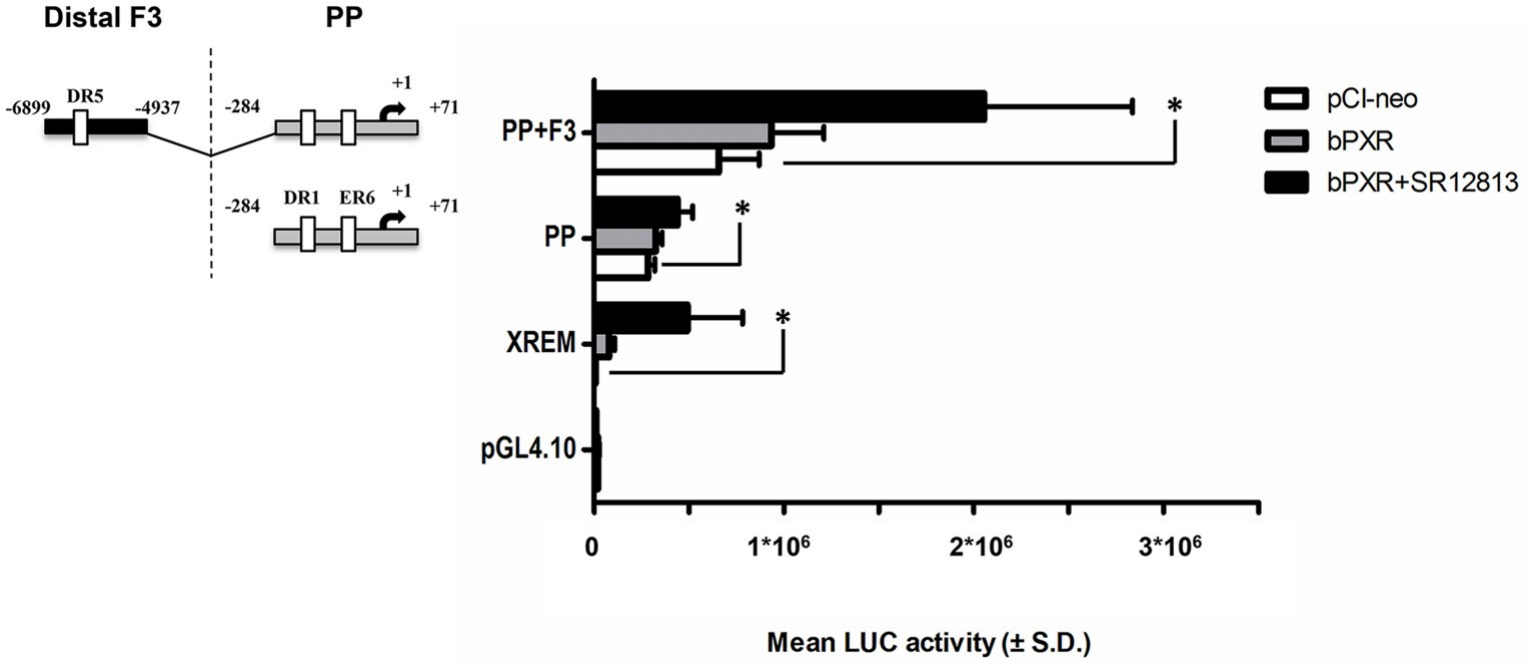
bPXR-mediated transactivation of the proximal promoter and the fragment 3 in *CYP3A28* promoter. The transactivation by bPXR of the most responsive fragments PP and F3 was evaluated. C3A cells were transfected with the control reporter pCMVβ (150 ng/well), each reporter plasmids or CYP3A4-XREM-luc (XREM, 50 ng/well) and either bPXR expression plasmids or pCI-neo empty vector (25 ng/well). After transfection, cells were treated with vehicle (0.1% DMSO) or SR12813 (10 μM) for 24 hours, and reporter activities were measured. Firefly luciferase activities were normalized with β-galactosidase activities. Data are expressed as mean luciferase activities ± SD (n = 3 or 4). Results shown are representative of 3 independent assays. Statistical significance *P* < 0.05: *, XREM, PP, PP+F3 with empty pCI-neo *vs*. ligand-treated bPXR.

To verify the consistency of *CYP3A28* PP (-284/+71 bp) and F3 (-6899/-4937bp) trans-activation by bPXR, the constructs PP and PP+F3 were extensively tested in C3A cells by co-transfecting the relevant expression vectors (empty pCI-neo, bPXR with or without SR12813 exposure). The C3A cells were chosen over HepG2 because of their better functionality in reporter gene-based assays [41]. The transfected bPXR increased the luciferase activity in XREM-3A4 construct, only slightly in PP and PP+F3, and had no effect on pGL4.10. The activation was further enhanced (~77-, 1.5- and 3.1-fold in XREM-3A4, PP and PP+F3, respectively) and reached the statistical significance by addition of SR12813 (*P* < 0.05; see Fig 4). The same assay using RIF exposure failed to activate any reporters confirming that RIF is not recognized by bPXR (S7 Table).

**Fig 5:**
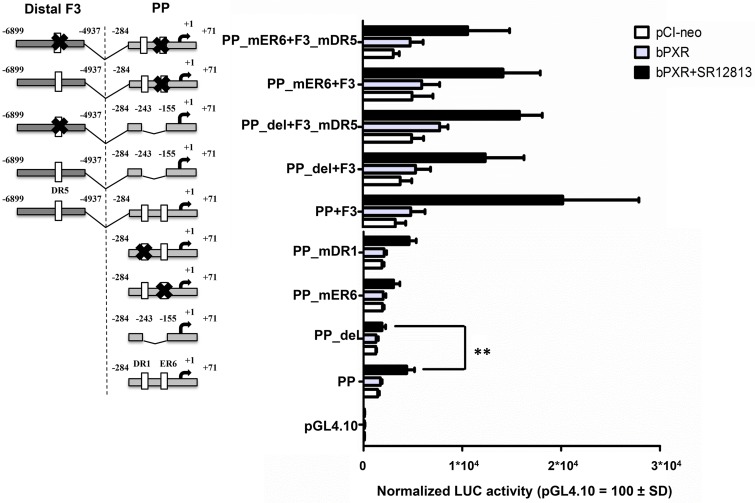
Identification of bPXR-responsive elements in the proximal promoter and fragment 3 in *CYP3A28* promoter. Several constructs were produced to study the binding elements identified in the *CYP3A28* proximal promoter (PP) and the contribution of the binding motif DR5 identified in F3. The parental PP was deleted of the whole putative region containing several TF binding-sites resulting in the PP_del; through site-directed mutagenesis the ER6 (PP_mER6) and DR1 (PP_mDR1) motifs were inactivated. The parental PP+F3 was deleted of the whole putative region containing several TF binding-sites resulting in the PP_del+F3; through site-directed mutagenesis the ER6 (PP_mER6+F3), the DR5 motif (PP+F3_mDR5 and PP_del+F3_mDR5) or both (PP_mER6+F3_mDR5) were inactivated. Details are reported in S1 File. Numbers indicate the positions relative to the transcriptional start site. C3A cells were transfected with the control reporter pCMVβ (150 ng/well), each reporter plasmid or CYP3A4-XREM-luc (XREM, 50 ng/well) and either bPXR expression plasmid or pCI-neo empty vector (25 ng/well). After transfection, cells were treated with vehicle (0.1% DMSO) or SR12813 (10 μM) for 24 hours, and reporter activities were measured. Firefly luciferase activities were normalized with β-galactosidase activities. Data are expressed as relative activities to those in pGL4.10 transfected cells (= 100) for each condition (pCI-neo empty or bPXR co-transfection). Data are the mean ± SD (n = 3 or 4). Results shown are representative of 3 independent assays. Statistical significance *P* < 0.01: **, PP with ligand-treated bPXR *vs*. PP_del with ligand-treated bPXR.

Next, the ER6, HNF-1 and HNF-4 elements located in the PP construct were deleted. This shorter PP (-284/-243 and -155/+71 bp; PP_del) displayed slightly decreased activity (-25%) in bPXR co-transfected cells, and significantly reduced activation (-57%) by SR12813 (*P* < 0.01; Fig 5). The mutagenesis of the ER6 motif (PP_mER6) did not alter the basal activity but led to a diminished SR12813 response (-30%), but not at the same extent than the PP_del (-57%). Surprisingly, the mutation of the predicted HNF-4 binding site (PP_mDR1) had no effect on either basal activity or the SR12813 response (Fig 5).

Similarly, the site-directed mutagenesis of the element DR5 (-5541/-5525 bp) present in F3 (-6899/-4937 bp) was performed to clarify its role in the observed response to bPXR and SR12813. The PP_del and the mutated PP_mER6 were used in combination to eliminate or reduce, respectively, the contribution by the intact PP. Both basal (empty pCI-neo) and bPXR-mediated activity of PP_del+F3mDR5 and PP_mER6+F3 were reduced by about 50%, indicating the presence of some important regulatory elements in F3. The mutation of DR5 (mDR5) reduced the reporter activity by 18% only in the context of PP_mER6 that contained a HNF-4 site but not in the context of PP_del that lacked both the HNF-4 and the proximal ER6 sites. This suggested that DR5 may have only a marginal role in bPXR-mediated activation that also depended on the intact *CYP3A28* PP.

### Role of bCAR in the transactivation of *CYP3A28* promoter

**Fig 6:**
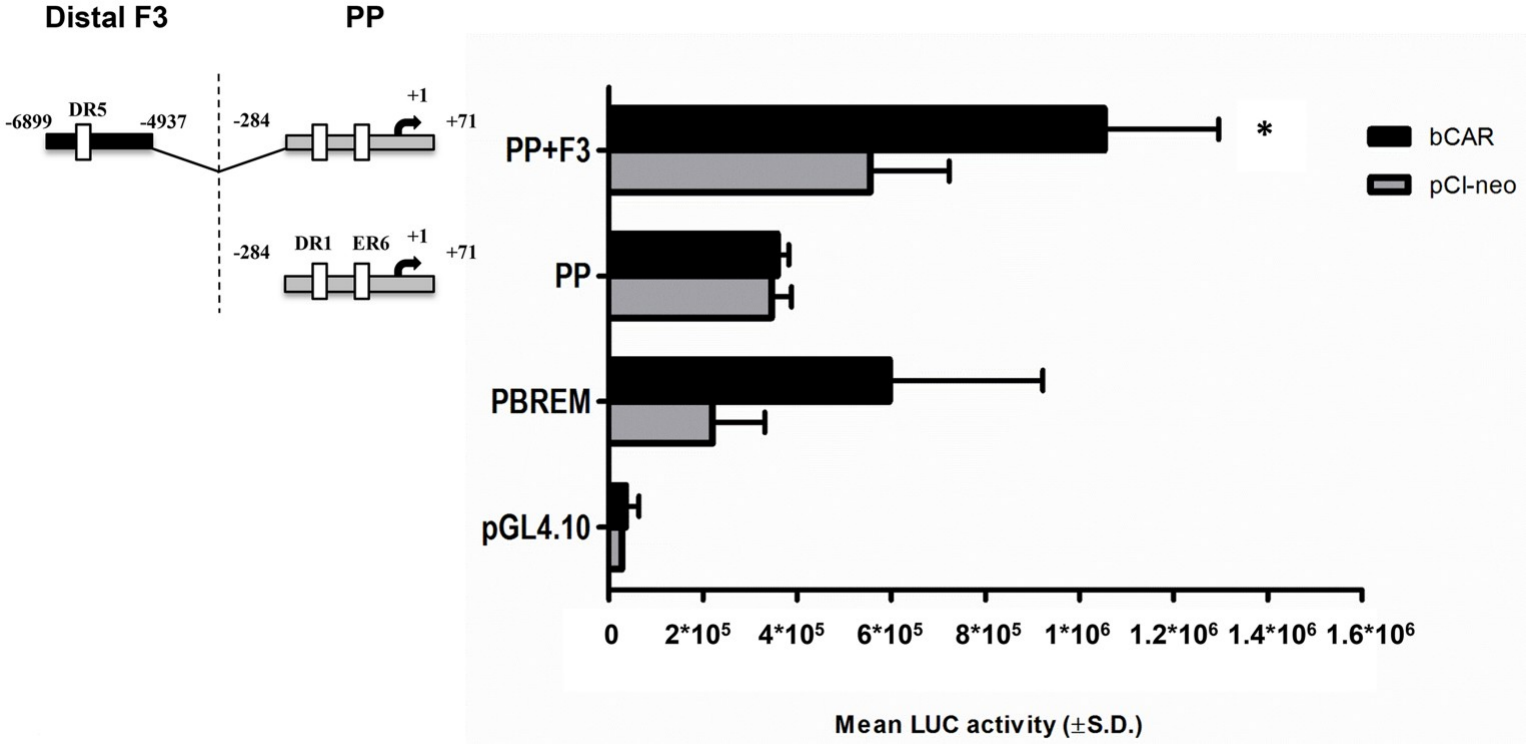
bCAR-mediated transactivation of the proximal promoter and the fragment 3 in *CYP3A28* promoter. The transactivation by bCAR of the most responsive fragments PP and F3 was evaluated. C3A cells were transfected with the control reporter pCMVβ (150 ng/well), each reporter plasmids or PBREM-tk-luc (PBREM, 50 ng/well) and either bCAR expression plasmids or pCI-neo empty vector (25 ng/well). After transfection, cells were treated with vehicle (0.1% DMSO) for 24 hours, and reporter activities were measured. Firefly luciferase activities were normalized with β-galactosidase activities. Data are expressed as mean luciferase activities ± SD (n = 3 or 4). Results shown are representative of 3 independent assays. Statistical significance *P* < 0.05: *, PP-F3 with empty pCI-neo *vs*. bCAR.

The ability of bCAR to transactivate the *CYP3A28* 5’-flanking regions was examined by transient transfection of C3A cells. As shown in Fig 6, bCAR activated the PBREM-tk-luc reporter. The PP did not show an increase in luciferase activity by co-transfection of bCAR. By contrast, co-transfection of bCAR enhanced the PP+F3 reporter activity by 1.4-fold (*P* < 0.05) as compared to the empty expression vector pCI-neo.

The same set of deleted and mutated constructs used to assess bPXR-dependency was employed to investigate bCAR-dependent activity (S3 Fig). However, no clear and statistically significant bCAR-dependency could be observed. The F3 fragment showed higher basal activity which seemed to be dependent on the presence of intact ER6 and DR5 motifs.

### *CYP3A28* induction in the BFH12 cell line

To assess *CYP3A28* inducibility in the recently available BFH12 cell line, cells were exposed to prototypical CYP3A inducers and their time- and dose-responses were determined.

*CYP3A28* mRNA expression was modestly but significantly induced by 10 μM RU486 after 6, 12 and 24 hours of exposure (*P* < 0.001) (S4 Fig, panels D, E, F). All other PXR ligands, used at the same concentration, did not significantly affect *CYP3A28* expression at any time point (S4 Fig). In particular, PCN and SR12813 only slightly increased (about 1.5-fold) *CYP3A28* mRNA, while RIF and DEX had no effect. The NRs CAR and RXRα, constitutively expressed in this bovine cell line, were not modulated by the treatment (S5 Fig, panels A and C). PXR showed a very low and variable constitutive expression in BFH12 cells (S5 Fig, panel B).

**Fig 7:**
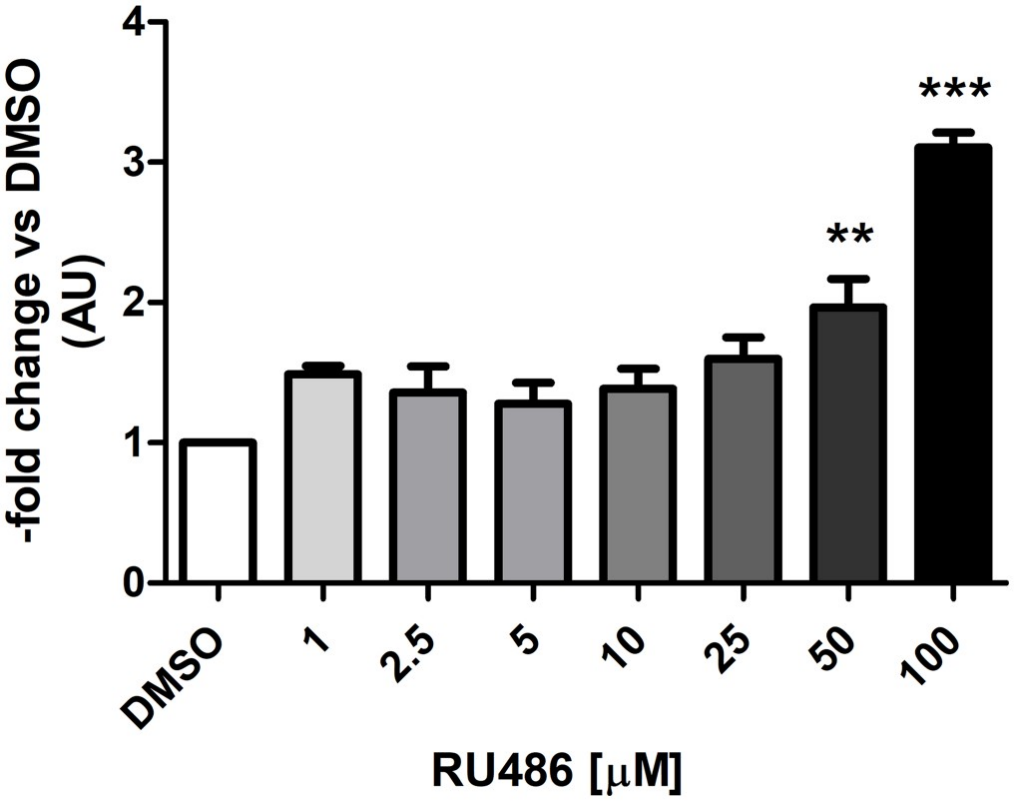
Induction of *CYP3A28* mRNA in BFH12 cells exposed to increasing concentrations of RU486 for 6 hours. BFH12 cells were treated with different concentrations of RU486 (1, 2.5, 5, 10, 25, 50 and 100 μM) for 6 hours, as described in Materials and Methods. The expression of *CYP3A28* was detected by qPCR in control (0.1% DMSO) and treated cells, using *RPLP0* as internal control gene. The relative expression of DMSO-treated cells was set to 1 and its value was used for the normalization of the other groups. Data are expressed as the mean ± SD of three independent experiments (arbitrary units, AU). Statistical analysis: ANOVA + Tukey’s post test. Significance was defined as: *P* < 0.01: **, DMSO *vs*. 50 μM RU486; *P* < 0.001: ***, DMSO *vs*. 100 μM RU486.

For dose responses, the incubation time was set at 6 hours, which showed a significant modulation of *CYP3A28* expression and thus, appropriate for ChIP experiments. Here, RU486 and SR12813 were selected because they were the most potent bPXR activators [9] modulating *CYP3A28* mRNA expression, while RIF was used as negative control. The selected concentrations (1–100 μM for RU486 and RIF, 1–25 μM for SR12813) were non-toxic after 6 hours of incubation (Alamar blue cytotoxicity test). A dose-dependent increase of *CYP3A28* mRNA expression was observed only for RU486 (Fig 7) at 50 μM (*P* < 0.01) and at 100 μM (~3-fold; *P* < 0.001). SR12813 and RIF did not affect the expression of *CYP3A28* mRNA (S6 Fig).

Among the selected bCAR activators (CITCO and FL81), only the highest tested concentration of FL81 (30 μM) significantly increased (*P* < 0.001) *CYP2B22* mRNA levels after 12 hours of incubation; however, no transcriptional effects on *CYP3A28* mRNA were ever noticed after 6 or 12 hours of incubation (S7 Fig).

### ChIP assays

Before the execution of ChIP investigations, the cross-reactivity of the anti-hCAR, anti-hPXR and anti-hRXR antibodies towards bovine proteins was assessed by immunoblotting. Each antibody recognized appropriately sized and localized proteins in the cytosolic and nuclear extracts of untreated bovine liver, as judged by positive controls from stable hCAR- and hPXR-expressing hepatic cell lines [41] (see S8 Fig).

**Fig 8:**
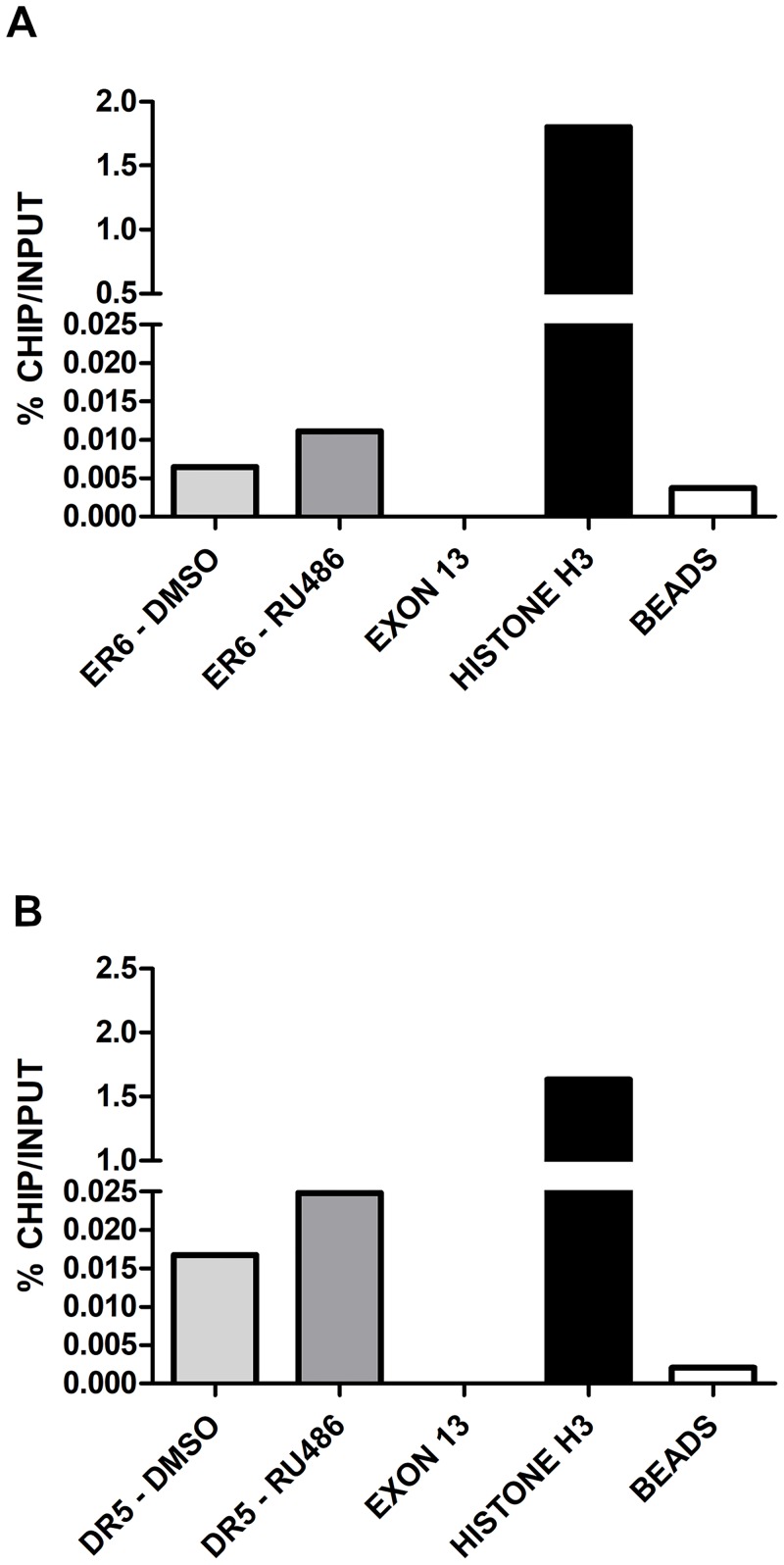
ChIP in control and treated BFH12 cells to quantify the binding of RXRα to ER6 and DR5 binding sites. BFH12 cells were exposed to 0.1% DMSO and 100 μM RU486 for 6 hours. Chromatin was isolated, subjected to ChIP using anti-human RXRα antibody and quantified by qPCR as described in S1 File. Results for ER6 and DR5 DNA regions are reported in panels A and B, respectively. Data are normalized to input DNA and expressed as % ChIP/input. The experiment was performed four times independently, and similar results were obtained. The data shown derived from a representative experiment. Chromatin samples from control cells immunoprecipitated with or without Histone H3 antibody are shown as Histone H3 and beads, respectively. A further negative control (exon 13), representing a *CYP3A28* DNA region without NR binding sites, is reported in the graph. In all experiments, negative and positive controls behaved as expected.

**Fig 9:**
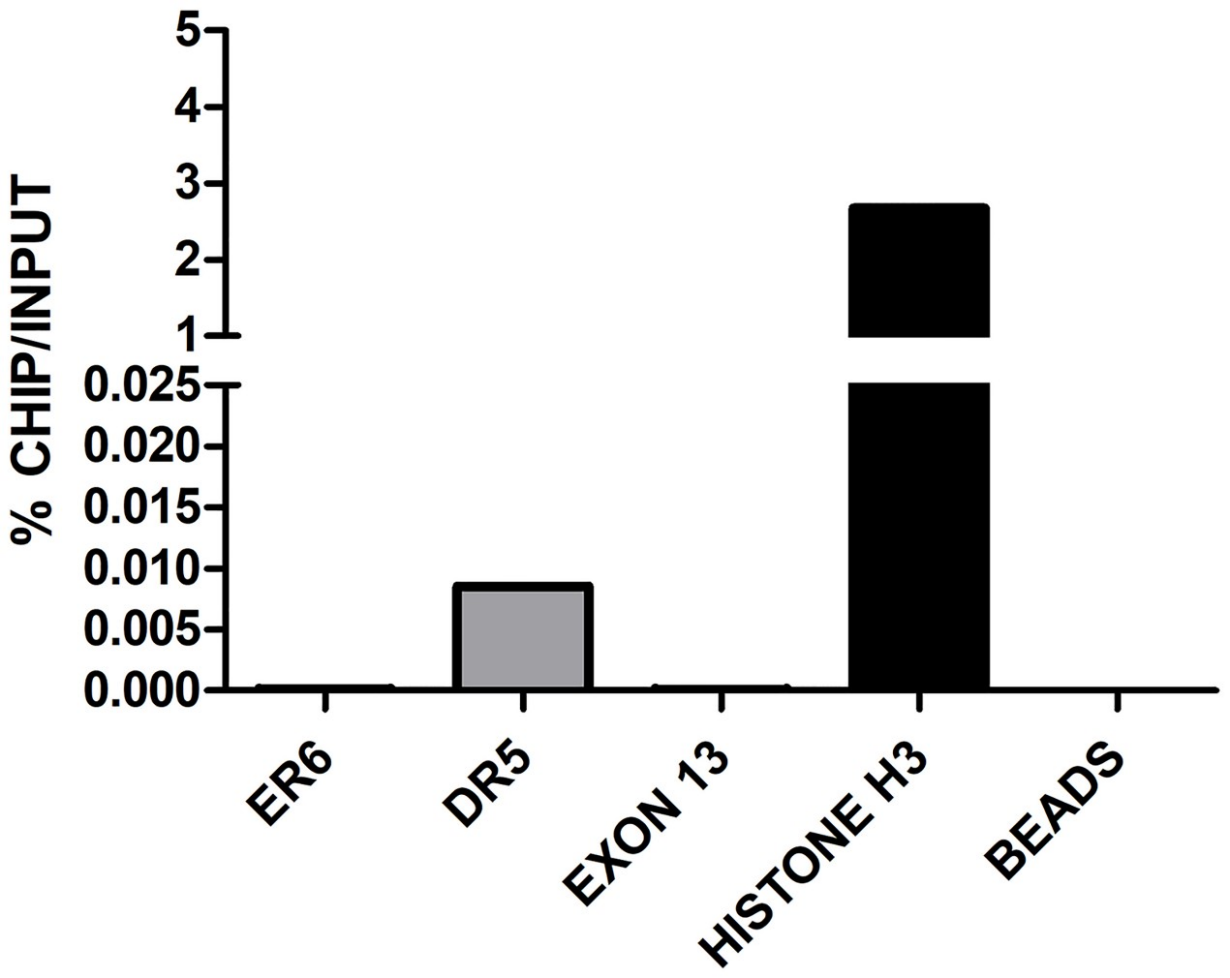
ChIP in control BFH12 cells to quantify the binding of CAR to ER6 and DR5 binding sites. BFH12 cells were exposed to 0.1% DMSO for 6 hours. Chromatin was then isolated, subjected to ChIP using anti-human CAR antibody and quantified by qPCR as described in S1 File. Results for both ER6 and DR5 DNA regions are reported. Data are normalized to input DNA and expressed as % ChIP/input. The experiment was performed four times independently, and similar results were obtained. The data shown derived from a representative experiment. Chromatin samples from control cells immunoprecipitated with or without Histone H3 antibody are shown as Histone H3 and beads, respectively. A further negative control (exon 13), representing a CYP3A28 DNA region without NR binding sites, is reported in the graph. In all experiments, negative and positive controls behaved as expected.

To confirm the involvement of PXR, CAR and RXRα in the transactivation of bovine *CYP3A28*, ChIP experiments on DMSO-, RU486- and FL81-treated BFH12 cells were done. In control conditions (0.1% DMSO), RXRα was bound to both ER6 and DR5 within the PP and the distal F3 fragment, respectively (Fig 8 and S9 Fig), suggesting the binding by a NR-RXR heterodimer, presumably by CAR or PXR. Using the anti-CAR antibody, an immunoprecipitation of DR5-containing DNA region was observed (Fig 9 and S10 Fig), consistent with our reporter gene assays and in alignment with RXRα binding. ChIP experiments using the anti-PXR antibody were not completely successful, as a very low immunoprecipitation of both ER6 and DR5 was obtained in control conditions, while only a qualitative increase in PXR binding to the DR5 motif was noticed following RU486 treatment (S11 Fig). These results are only suggestive of bPXR recognizing both ER6 and DR5 binding sites, but the low binding was likely due to the very low constitutive expression of PXR in BFH12 cells. In fact, the incubation of BFH12 cells with 100 μM RU486 showed a trend indicative of a mild increase in the recruitment of RXRα (approximately 1.5–3-fold) to either the ER6 motif (Fig 8A and S9 Fig, panel A and B) than the DR5 motif in the distal F3 fragment (Fig 8B and S9 Fig, panel C and D) in all experiments even though there was a significant variation among individual reactions. These qualitative results indirectly suggest the involvement of PXR/RXR heterodimer in *CYP3A28* transactivation by recognition of both ER6 and DR5 binding motifs. Furthermore, the increase in the binding of RXRα to the proximal ER6 was consistent with *CYP3A28* mRNA induction.

Overall, the specific antibody enrichment, detected in ChIP investigations and expressed as a percentage of input, was extremely low (in the range of 0.01%), probably due either to the low constitutive expression of NRs in BFH12 cells or the weak responsiveness of this fetal cell line to prototypical CYPs inducers, as described above.

## Discussion

In the present study, NR-mediated regulation of bovine *CYP3A28* transcription was investigated through DNA re-sequencing, *in silico* prediction of NR/TF binding sites, gene reporter assays of wild-type and mutated promoter fragments, induction studies in a bovine-derived hepatic cell line and ChIP assays.

We showed that the bPXR is involved in the transcriptional regulation of *CYP3A28* expression via an ER6-type motif at -156 bp within the PP. This ER6 element is conserved among most of the *CYP3A* promoters in primates and other placental mammals [46]. It conferred RIF responsiveness to a heterologous reporter in rabbit hepatocytes [47] and binding of PXR to the ER6 motif activated transcription of *CYP3A4* reporters [48–50]. *CYP3A4* activation is also dependent upon distal enhancer modules XREM and CLEM [29,38,51]. Our *in silico* analysis did not identify XREM and CLEM modules within the 10 kbp *CYP3A28* promoter, although CAR- and PXR-responsive upstream regions were present (fragment F3). In a bioinformatic study using human *CYP3A4* sequences as a query [46], the proER6 and the CLEM sequences were found on cow *CYP3A*. In similar analyses, we found a CLEM-like sequence located ~34 kb upstream of the *CYP3A24* sequence or ~77 kb upstream of the *CYP3A38* gene, but not in the vicinity of the *CYP3A28* promoter. Therefore, the lack of CLEM that could synergize with the proximal ER6 element might explain its modest responsiveness to bCAR or to its moderate induction *in vivo* [14].

When the *CYP3A28* PP (bases -284 to +71) was activated by the ectopically expressed bPXR, the reporter gene expression increased about 2- to 3-fold. The extent of activation was about the same magnitude as with the human *CYP3A4* ER6 element [48]. RIF failed to activate *CYP3A28* PP, in accordance with the recent profiling of bPXR ligand-binding domain [9].

The distal fragment F3 (-6899/-4937 bp) conferred a higher basal activity and modest 2-fold responsiveness to bPXR to the *CYP3A28* PP construct. A putative DR5 element (-5525 bp) and several HNF-3, HNF-4 and C/EBPα binding sites could contribute to F3 bPXR and bCAR responsiveness, because also DR5 elements can serve as functional sites for CAR and PXR [49,52,53].

The additional NR-like elements elsewhere in the promoter (fragments lnPP, F5) were also surrounded by LETF sites, but we did not detect any responsiveness to either bCAR or bPXR in co-transfection analyses. They cannot be completely ruled out, because reporter plasmids are not organized in the same fashion as in the natural chromatin context [54].

The upstream region F2 (-4998/-3133 bp) was found to suppress the basal and bPXR- and bCAR-dependent reporter activity, even though it contained HNF-3, HNF-4 and CAR binding sites. Similar clusters of HNF-4 sites are present in the human *CYP3A4* gene, and at least in case of CLEM, the proximal *CYP3A4* promoter activity was decreased [55]. Although HNF-3 proteins are usually associated with activation [56], they can also repress transcription [57,58]. Therefore, the observed negative regulation requires further investigations, preferably with a natural *CYP3A28* promoter context.

Next, we focused on two NR site-containing regions that increased the *CYP3A28* reporter gene activity (fragments PP and F3). The deletion of the ER6, HNF-1 and HNF-4 binding sites in the *CYP3A28* PP eliminated the bPXR-mediated activation by SR12813. Upon mutagenesis, the ER6 motif but not the HNF-4 site appeared to be critical. This suggested that the ligand-activated bPXR is able to recognize ER6 and that HNF-1 could participate in bPXR-mediated activation. Recently, an HNF-1 binding site located at -32 kbp upstream of the rat *CYP3A1* was proved to act synergistically with DR4 and DR2 motifs to mediate the transactivation by rat CAR [59].

To confirm the relevance of the ER6 motif, ChIP assays were performed in BFH12 cells treated with RU486, the most potent inducer of *CYP3A28* mRNA and a strong activator of bPXR [9]. Among other established CYP3A inducers, DEX, PCN and RIF did not induce bovine *CYP3A28* mRNA, which was consistent with their poor ability to activate bPXR [9] or weak inductive effect of DEX *in vivo* and in liver slices [17,25,60].

ChIP assays in DMSO- and RU486-treated cells showed that RU486 weakly increased the recruitment of RXRα, the obligate heterodimer partner for CAR and PXR, for the binding to ER6. It was not possible to reliably detect binding of PXR, probably due to its low expression in BFH12 cells and its modest cross-reactivity with the anti-human PXR antibody. The contribution of HNF-4 in the PP activation was not evaluated by ChIP, because of its slight mRNA expression in BFH12 cells [28]. Conversely, the binding of HNF-1α and HNF-1β to this DNA was investigated but gave no conclusive outcome, yet again for the low constitutive expression of these LETFs in BFH12 cells [28].

Concerning DR5, the binding site located at -5525 bp in F3, luciferase assays did not clearly identify it as the element responsible for the bPXR- or bCAR-dependent activation. It is thus possible that many other LETF sites in this 1.5-kbp fragment cooperate with either DR5 or the proximal ER6 to confer NR responsiveness. Therefore, a deeper characterization of the fragment F3 is required. Nevertheless, ChIP experiments suggested that DR5 motif could recruit both CAR and PXR. DR5 seemed the main DNA binding site recognized by CAR, and the immunoprecipitation of both PXR and RXRα was qualitatively and slightly increased by RU486 treatment. Unfortunately, FL81 did not affect CAR-mediated DR5 immunoprecipitation, probably for two reasons. First, its rather modest increase (1.4-fold) of *CYP3A28* and *CYP2B22* mRNA expression in BFH12 cells, comparatively lower than that elicited by FL81 in human hepatocytes [34]; second, the high constitutive activity of CAR in the absence of added ligands [34], as demonstrated also in the bovine species [9].

The limitations of the present study are mainly attributable to ChIP assay results. First, a low specific antibody enrichment of ER6 and DR5 regions was generally obtained. This result could be justified by the fact that antibodies specific to bovine NRS were not available; moreover, the present bovine liver cell line (the only available at present) probably is not the optimal *in vitro* model because it does not express the whole set of LETFs and CYPs and does not fully respond to prototypical CYP inducers. As an example, the CYPs induction by PB, a hallmark of CAR-mediated regulation *in vivo* in calves [14,15], in rodents and humans (reviewed by [61]) could not be reproduced in BFH12 cells (S8 Table). Additionally, bCAR (CITCO and FL81) and bPXR activators (RU486, SR12813) previously identified by reporter gene assays [9] did not affect or only weakly increased *CYP3A28* mRNA expression. Secondly, as negative controls, we used no-antibody control (beads only) and a negative locus (*CYP3A28* exon 13) as already reported in previous publications [62–65] instead of IgG control (beads with an isotype matched control immunoglobulin) to evaluate the background and confirm the absence of unspecific binding of target proteins to beads.

## Conclusions

In summary, we have localized two regions potentially responsive to bPXR in the *CYP3A28* gene promoter, the proximal ER6 motif and a distal element F3. We hypothesized after protein/DNA interaction investigations that bCAR, despite the weak response in reporter assays, is recruited to the DR5 element in the distal F3 fragment. However, further molecular studies are needed to confirm ChIP results here obtained.

Overall, this work represents the first mechanistic study on bovine *CYP3A* regulation and it opens fresh avenues for more detailed analysis.

## Supporting information

S1 File Supplemental Material and Methods and References. (PDF)

S1 Table Primers used for the *ex novo* sequencing of bovine *CYP3A28* promoter region. (PDF)

S2 Table Oligonucleotide sequences for the amplification of the bovine *CYP3A28* promoter through long (ln) PCR reactions. (PDF)

S3 Table Primer sequences used for nested PCR reactions of the bovine *CYP3A28* promoter. (PDF)

S4 Table Oligonucleotides used for the internal site-directed mutagenesis of transcription factor binding sites. (PDF)

S5 Table Oligonucleotides used in the inverse PCR procedure. (PDF)

S6 Table Oligonucleotides used in qPCR ChIP. (PDF)

S7 Table bPXR-mediated transactivation of *CYP3A28* proximal promoter (PP) and fragment 3 (F3) using rifampicin (RIF). (PDF)

S8 Table Modulation of *CYP2B22* mRNA in BFH12 cells exposed for 6 hours to different concentrations of phenobarbital (PB). (PDF)

S1 Fig Schematic organization of the bovine *CYP3A* locus. Four *CYP3A* coding genes are known: *CYP3A28*, *CYP3A38*, *CYP3A48* and the predicted *CYP3A24*. The GenBank IDs and nomenclature are displayed together with the nomenclature proposed by [27].
(PDF)

S2 Fig Resolution of the two encountered gaps in the bovine *CYP3A28* promoter region. The re-sequencing of *CYP3A28* promoter region was able to solve the first gap starting at –1492 bp revealing a sequencing artefact (A), as well as the second gap at base –2988 with a new genomic sequence (B). Two of the five single nucleotides polymorphisms (-2899T>G and -2981_-2979insA) are here shown circled.
(PDF)

S3 Fig Identification of bCAR-responsive elements in the proximal promoter and fragment 3 in *CYP3A28* promoter. Several constructs were produced to study the binding elements identified in the *CYP3A28* proximal promoter (PP) and the contribution of the binding motif DR5 identified in F3. The parental PP was deleted of the whole putative region containing several TF binding-sites resulting in the PP_del; through site-direct direct mutagenesis the ER6 (PP_mER6) and DR1 (PP_mDR1) motifs were inactivated. The parental PP+F3 was deleted of the whole putative region containing several TF binding-sites resulting in the PP_del+F3; through site-direct direct mutagenesis the ER6 (PP_mER6+F3), the DR5 motif (PP+F3_mDR5 and PP_del+F3_mDR5) or both (PP_mER6+F3_mDR5) were inactivated. Details are reported in S1 File. Numbers indicate the positions relative to the transcriptional start site. C3A cells were transfected with the control reporter pCMVβ (150 ng/well), each reporter plasmid or PBREM-tk-luc (50 ng/well) and either bCAR expression plasmid or pCI-neo empty vector (25 ng/well). After transfection, cells were treated with vehicle (0.1% DMSO) for 24 hours, and reporter activities were measured. Firefly luciferase activities were normalized with β-galactosidase activities. Data are expressed as relative activities to those in pGL4.10 transfected cells (= 100) for each condition (pCI-neo empty or bCAR co-transfection). Data are the mean ± SD (n = 3 or 4). Results shown are representative of three independent assays.
(PDF)

S4 Fig Induction of *CYP3A28* mRNA in BFH12 cells exposed for 0, 1, 3, 6, 12 and 24 hours to five prototypical CYP3A inducers. BFH12 cells were treated with different CYP3A inducers (DEX, PCN, RIF, RU486 and SR12813) at the fixed concentration 10 μM for 0 (A), 1 (B), 3 (C), 6 (D), 12 (E) and 24 (F) hours. The expression of *CYP3A28* was detected by qPCR in control (0.1% DMSO) and treated cells, using *RPLP0* as internal control gene. The relative expression of DMSO-treated cells was set to 1 and its value was used for the normalization of the other groups. Data are expressed as the mean ± SD of three independent experiments (arbitrary units, AU). Statistical analysis: ANOVA + Tukey’s post test. Significance was defined as *P* < 0.05: *; *P* < 0.01: **; *P* < 0.001: ***.
(PDF)

S5 Fig Induction of CAR, PXR, RXRα mRNAs in BFH12 cells exposed for 0, 1, 3, 6, 12 and 24 hours to five prototypical CYP3A inducers. BFH12 cells were treated with different CYP3A inducers (PCN, RU486, SR12813, DEX and RIF) at the fixed concentration 10 μM for 0, 1, 3, 6, 12 and 24 hours. The expression of *CAR* (A), *PXR* (B) and *RXRα* (C) was detected by qPCR in control (0.1% DMSO) and treated cells, using *RPLP0* as internal control gene. The relative expression of DMSO-treated cells was set to 1 and its value was used for the normalization of the other groups. Data are expressed as the mean ± SD of three independent experiments (arbitrary units, AU). Statistical analysis: ANOVA + Tukey’s post test.
(PDF)

S6 Fig Induction of *CYP3A28* mRNA in BFH12 cells exposed to increasing concentrations of SR12813 and RIF for 6 hours. BFH12 cells were treated with different concentrations of SR12813 (1, 2.5, 5, 10, 25 μM) and RIF (1, 2.5, 5, 10, 25, 50 and 100 μM) for 6 hours, as described in Materials and Methods. The expression of *CYP3A28* was detected by qPCR in control (0.1% DMSO) and treated cells, using *RPLP0* as internal control gene. The relative expression of DMSO-treated cells was set to 1 and its value was used for the normalization of the other groups. Data are expressed as the mean ± SD of two independent experiments (arbitrary units, AU). Statistical analysis: ANOVA + Tukey’s post-test.
(PDF)

S7 Fig Induction of *CYP3A28* and *CYP2B22* mRNA in BFH12 cells exposed for 6 and 12 hours to FL81. BFH12 cells were exposed to different concentrations (1, 3, 10 and 30 μM) of the bCAR activator, FL81, for 6 and 12 hours. The expression of *CYP3A28* (A, B) and *CYP2B22* (C, D) mRNA was detected by qPCR in control (0.1% DMSO) and treated cells, using *RPLP0* as internal control gene. The relative expression of DMSO-treated cells was set to 1 and its value was used for the normalization of the other groups. Data are expressed as the mean ± SD of three independent experiments (arbitrary units, AU). Statistical analysis: ANOVA + Tukey’s post-test. Significance was defined as *P* < 0.01: **; *P* < 0.001: ***. A, C: 6 hours of incubation; B, D: 12 hours of incubation.
(PDF)

S8 Fig CAR, PXR and RXR immunoblotting analysis of subcellular fractions isolated from untreated bovine liver. Bovine liver cytosolic and nuclear extracts were isolated according to Renisalo et al. (2012) with minor modifications. Proteins (30 μg) were subjected to immunoblotting analysis following the protocol previously published by [14]. Membranes were firstly probed with anti-human CAR (1:1000 final dilution), anti-human PXR (1:1000) and anti-human RXR (1:1500) polyclonal antibodies and then with a peroxidase-conjugated goat anti-rabbit IgG (Chemicon International; 1:6000 final dilution). As positive control, total proteins isolated from C3A cells stably transfected with hCAR or hPXR [41] was used. 1: molecular weight marker (ChemiBlot Molecular Weight Marker, Millipore); 2: C3A cells transfected with hPXR or hCAR, total proteins; 3: bovine liver tissue, cytosol; 4: bovine liver tissue, nuclear fraction.
(PDF)

S9 Fig ChIP in control and treated BFH12 cells to quantify the binding of RXRα to ER6 and DR5 binding sites. BFH12 cells were exposed to 0.1% DMSO and 100 μM RU486 for 6 hours. Chromatin was isolated, subjected to ChIP using anti-human RXRα antibody and quantified by qPCR as described in S1 File. Results for ER6 and DR5 DNA regions are reported in panels A-B and C-D, respectively. The data shown derived from two further independent experiments; they are normalized to input DNA and expressed as % ChIP/input. The experiment was performed four times independently, and similar results were obtained. Chromatin samples from control cells immunoprecipitated with or without Histone H3 antibody are shown as Histone H3 and beads, respectively. A further negative control (exon 13), representing a *CYP3A28* DNA region without NR binding sites, is reported in the graph. In all experiments, negative and positive controls behaved as expected.
(PDF)

S10 Fig ChIP in control BFH12 cells to quantify the binding of CAR to ER6 and DR5 binding sites. BFH12 cells were exposed to 0.1% DMSO for 6 hours. Chromatin was then isolated, subjected to ChIP using anti-human CAR antibody and quantified by qPCR as described in S1 File. Results for both ER6 and DR5 DNA regions are reported. The data shown derived from two further independent experiments (panels A, B); they are normalized to input DNA and expressed as % ChIP/input. The experiment was performed four times independently, and similar results were obtained. Chromatin samples from control cells immunoprecipitated with or without Histone H3 antibody are shown as Histone H3 and beads, respectively. A further negative control (exon 13), representing a *CYP3A28* DNA region without NR binding sites, is reported in the graph. In all experiments, negative and positive controls behaved as expected.
(PDF)

S11 Fig ChIP in control and treated BFH12 cells to quantify the binding of PXR to ER6 and DR5 binding sites. BFH12 cells were exposed to 0.1% DMSO and 100 μM RU486 for 6 hours. Chromatin was isolated, subjected to ChIP using anti-human PXR antibody and quantified by qPCR as described in Material and Methods. Results for ER6 and DR5 DNA regions are reported in panel A and B, respectively. Data are normalized to input DNA and expressed as % ChIP/input. The experiment was performed four times independently, and similar results were obtained. The data shown derived from a representative experiment. Chromatin samples from control cells immunoprecipitated with or without Histone H3 antibody are shown as Histone H3 and beads, respectively. A further negative control (exon 13), representing a *CYP3A28* DNA region without NR binding sites, is reported in the graph. In all experiments, negative and positive controls behave as expected.
(PDF)

## Abbreviations

AU: arbitrary units
C/EBPα: CCAAT/enhancer binding protein-α
CAR: constitutive androstane receptor
ChIP: chromatin immunoprecipitation
CITCO: 6-(4-chlorophenyl)imidazo[2,1-*b*][1,3]thiazole-5-carbaldehyde O-(3,4-dichlorobenzyl)oxime
CLEM: constitutive liver enhancer module
CYP: cytochrome P450
DBD: DNA binding domain
DEX: dexamethasone
DMSO: dimethyl sulfoxide
DR5: direct repeat motif with a 5-nucleotide spacer
ER6: everted repeat motif with a 6-nucleotide spacer
F: forward
FBS: fetal bovine serum
FL81: 5-(3,4-dimethoxybenzyl)-3-phenyl-4,5-dihydroisoxazole
HNF: hepatocyte nuclear factor
LBD: ligand binding domain
LETF: liver-enriched transcription factor
lnPCR: long PCR
lnPP: long proximal promoter
NR: nuclear receptor
PB: phenobarbital
PBS: phosphate buffer saline
PCN: pregnenolone 16α-carbonitrile
PP: proximal promoter
PXR: pregnane X receptor
qPCR: quantitative real-time PCR
R: reverse
RIF: rifampicin
RU486: mifepristone
RXRα: retinoid X receptor α
SR12813: 4-[2,2-bis-(diethoxyphosphoryl)ethenyl]-2,6-ditert-butylphenol
TF: transcription factor
XREM: xenobiotic-responsive enhancer module
