# Supplementary material for: DNA elements for constitutive androstane receptor- and pregnane X receptor-mediated regulation of bovine CYP3A28 gene
Source: PLoS One. 2019 Mar 25;14(3):e0214338. doi: 10.1371/journal.pone.0214338 (PMC6433341; doi:10.1371/journal.pone.0214338)
Supplement: S1 File — (PDF) [file pone.0214338.s001.pdf]

Title: DNA Elements for Constitutive Androstane Receptor- and Pregnane X Receptor-mediated Regulation of Bovine *CYP3A28* Gene

Authors: Mery Giantin, Jenni Küblbeck, Vanessa Zancanella, Viktoria Prantner, Fabiana Sansonetti, Axel Schoeniger, Roberta Tolosi, Giorgia Guerra, Silvia Da Ros, Mauro Dacasto, Paavo Honkakoski

Journal: Plos One

Supplemental Information File

Supplemental Materials and Methods

Re-sequencing of the *Bos taurus CYP3A28* gene promoter

CYP3A28 promoter sequence analysis

Reporter vectors

Site-directed mutagenesis of TF binding sites

Inverse PCR-based deletion of TF binding sites

Chromatin immunoprecipitation (ChIP) assay

References cited in the Supplemental Materials and Methods section

Supplemental Materials and Methods

Re-sequencing of the *Bos taurus CYP3A28* gene promoter

Bos taurus liver tissue was collected at a commercial slaughterhouse from male Piedmontese beef cattle, immediately snap-frozen and stored at -80°C until use. The preparation of total DNA from liver was performed using the Invisorb Spin Tissue Mini Kit (STRATEC Molecular, Berlin, Germany). Purified DNA was checked for its quantity and quality with NanoDrop ND-1000 (Thermo Fisher Scientific, Waltham, Massachusetts, USA) and stored at -20°C. Approximately 10 kbp of *CYP3A28* gene promoter was sequenced *ex novo* from a selected sample. The primers were designed on the published Hereford genome assembly UMD 3.1 (GenBank Assembly ID: GCA_000003055.3) and their sequences are listed in S1 Table. Phusion High-Fidelity DNA Polymerase (Thermo Fisher Scientific) was used to carry out specific PCR amplifications (T-Personal Thermocycler, Biometra, Göttingen, Germany). Partially overlapping PCR products (400 - 1300 bp) were initially checked by 1.5% agarose gel electrophoresis before sequencing at BMR Genomics laboratories (Padua, Italy). Each promoter fragment was sequenced at least three times from independent PCR reactions. The software DNA Star SeqMAN II (DNA Star Inc., Madison, WI) was used to assemble the final promoter contig. The updated *CYP3A28* promoter sequence was submitted to GenBank (NCBI) with the accession number KU696412.

CYP3A28 promoter sequence analysis

Putative binding sites for PXR, CAR, HNF-1, HNF-3, HNF-4 and C/EBPα were predicted within the 10368 bp of the bovine *CYP3A28* promoter. The NR binding sites were located by NUBIscan (Podvinec et al., 2002) and NHR scan (Sandelin and Wasserman, 2005) algorithms. NUBIscan search was customized for the identification and scoring of two specific motifs recognized by PXR-RXR and CAR-RXR heterodimers, ER6-9 and DR3-5 motifs, with a threshold

of Z > 8.0. NHR scan searches were performed using the default settings. MatInspector (Release Professional 8.0.5: Cartharius et al., 2005) was applied to identify the retinoid X receptor (RXRα; NR2B1), HNF-3, HNF-4 and C/EBP binding sites, with threshold of 100% in core similarity and a matrix similarity value according to the optimized matrix threshold for the binding site. Match (1.0 Public) software employs library of positional weight matrices from TRANSFAC Public 6.0 to search for potential binding sites (Kel et al., 2003). The tool was interrogated for high quality and liver-specific matrices in vertebrates, with a chosen cut-off to minimize false positive results. This cut-off should be useful for searching the most promising TF binding sites in extended genomic DNA sequences.

Reporter vectors

Bovine genomic DNA was used to generate four long PCR (lnPCR) fragments by using TaKaRa LA Taq Hot Start DNA polymerase (TaKaRa Biotechnology Co., Otsu, Japan) covering the whole *CYP3A28* promoter: lnPCR#1 from -2662 to +387 bp, lnPCR#2 from -7335 to +387 bp, lnPCR#3 from -10651 to -2078 bp and lnPCR#4 from -10651 to -7096 bp (see S2 Table for primers). The amplified products were purified and used as template for nested PCR performed with the Q5 High-Fidelity DNA Polymerase (New England BioLabs, Ipswich, MA). Seven products containing different putative binding sites were generated: the proximal promoter (PP) from -284 to +71 bp; the long PP (lnPP) from -1359 to +71 bp; the fragment F1 from -3133 to -262 bp; the fragment F2 from -4998 to -3133 bp; the fragment F3 from -6899 to -4937 bp; the fragment F4 from -8314 to -6742 bp and the fragment F5 from -10368 to -8259 bp. The PP and lnPP fragments were double-digested with *Bgl*II and *Hind*III (Thermo Fisher Scientific) and ligated to the *Bgl*II/*Hind*III-cut pGL4.10[luc2] vector (Promega, Madison, WI). Several plasmids, purified with Qiagen's QIAprep Spin Miniprep (Qiagen, Hilden, Germany) were sequenced by Macrogen Europe laboratories (Amsterdam, The Netherlands) to confirm the successful cloning. Primer sequences and restriction enzymes' details are provided in S3 Table. All constructs were verified by

DNA sequencing. The PP construct was used as a minimal promoter into which upstream fragments were cloned, giving rise to promoter fragments PP+F1, PP+F2, PP+F3, PP+F4 and PP+F5.

Site-directed mutagenesis of TF binding sites

Site-directed mutagenesis was performed for the ER6 motif and HNF-4 binding site (DR1) identified in PP and for the DR5 motif in fragment F3. A series of different constructs are represented in the Figures 4 and 6. The second and the fifth base (5'→3') of each AGGTCA-like motif are most conserved, and they were mutated into the least represented nucleotide (Fang et al., 2012) and accompanied by a diagnostic restriction enzyme site for detection of successful mutagenesis (S5 Table). Using Pfu DNA Polymerase (Thermo Fisher Scientific), 10 ng of plasmid DNA were amplified with the mutagenic primers according to the manufacturer's instructions. The amplified product was then digested by *Dpn*I restriction endonuclease (Thermo Fisher Scientific) to remove the parent methylated and hemimethylated DNA. Finally, mutated plasmid DNAs were transformed into DH5α *E. coli* competent cells (New England BioLabs, Ipswich, MA) and then several colonies have been picked up and plasmid DNA was isolated. The plasmid DNA was later screened first by restriction digestion and agarose gel electrophoresis, followed by sequencing.

Inverse PCR-based deletion of TF binding sites

The ER6, HNF-1 and HNF-4 binding sites were deleted from PP and PP+F3 promoter constructs, and two "shorter" plasmids called PP_del and PP_del+F3 were generated by the inverted PCR-based method (Erster et al., 2010) (see S4 Table for primers) with the aid of Q5 High-Fidelity DNA Polymerase (New England BioLabs). The linearized fragments with the desired deletion were re-circularized by intramolecular ligation with T4 ligase (New England BioLabs), isolated and verified by sequencing.

Chromatin immunoprecipitation (ChIP) assay

After BFH12 cells incubation with RU486, FL81 and DMSO and cells detachment, nuclear proteins were cross-linked to DNA by adding formaldehyde to a final concentration of 1% for 10 min. Cross-linking was stopped by adding glycine to a final concentration of 0.125 M and incubating at room temperature for 5 min on a rocking platform. The medium was removed and cells were washed in ice-cold PBS. After centrifugation, nuclei were isolated resuspending the cell pellets in a lysis buffer (50 mM Tris-HCl, 150 mM NaCl, 5 mM EDTA, 1% Triton-X, pH 8) containing protease inhibitors for 30 min. Nuclei were then resuspended in a shearing buffer (50 mM Tris-HCl, 1 mM EDTA, 0.5% SDS, pH8) and chromatin was fragmented using the Microson Ultrasonic Cell Disruptor (Heat Systems, Germany) at power 30% for 18 pulses (20 s each followed by 30 s of rest), to produce fragments of 300-1000 nucleotides. The sonicated samples were centrifuged at 14.000xg at 4°C for 10 min to clear the supernatants. A small aliquot (25 µL) was reverse cross-linked to allowing DNA quantification by Qubit fluorometer and quality control by agarose gel electrophoresis and used as input (a part of the sample not subjected to ChIP); the remaining chromatin was stored at -80°C.

ChIP assays were performed using the following antibodies: rabbit anti-CAR (Abcam, ab83850), anti-PXR (Abcam, ab192579) and anti-RXR (Santa Cruz Biotechnology D-20, sc-553). Anti-histone H3 (Abcam, ab1791) and beads in absence of any antibody were used as positive and negative ChIP controls, respectively. Fifty mg of Dynabeads Protein G (Thermo Fisher Scientific) were incubated first with 4 µg of the specific antibody (5 µg for CAR and PXR) for 2 hours at 4°C on a rotator and then with albumin solution 5 µg/mL for further 2 hours. For the subsequent immunoprecipitation, the same beads were then incubated with 10 µg of pre-cleared chromatin overnight at 4°C on a rotator and then washed with low-salt, high-salt and lithium chloride-containing washing buffers. The precipitated DNA was finally eluted twice in 260 µL of elution buffer (0.1 M $NaHCO_3$, 1% SDS) on a rotating wheel for 30 min at 65°C, reverse cross-linked by overnight incubation at 65°C, extracted using a classical phenol/chloroform precipitation method

and subjected to qPCR with the primers listed in S6 Table. Primers were designed to span the elements of interest (ER6 in the proximal and DR5 in the F3 fragments). The ChIP assay will cover also binding sites for HNF-1 and HNF-4 due to their proximity to the ER6 motif. As negative control, a *CYP3A28* coding region (exon 13) was selected. qPCR of immune-precipitated DNA was performed as reported in the previous paragraph, using 300 nM final concentration of each primer, 1 ng of input or 2 uL of undiluted precipitated DNA. Finally, the expression was normalized to the percentage of input sample. At least three independent experiments were performed.

References cited in the Supplemental Materials and Methods section

Cartharius K, Frech K, Grote K, Klocke B, Haltmeier M, Klingenhoff A, et al. MatInspector and beyond: promoter analysis based on transcription factor binding sites. Bioinformatics 2005;21: 2933-2942.

Erster O and Liscovitch M. A modified inverse PCR procedure for insertion, deletion, or replacement of a DNA fragment in a target sequence and its application in the ligand interaction scan method for generation of ligand-regulated proteins. Methods Mol Biol. 2010;634: 157-174.

Fang B, Mane-Padros D, Bolotin E, Jiang T, Sladek FM. Identification of a binding motif specific to HNF4 by comparative analysis of multiple nuclear receptors. Nucleic Acids Res. 2012;40: 5343-5356.

Kel AE, Gössling E, Reuter I, Cheremushkin E, Kel-Margoulis OV, Wingender E. MATCH: A tool for searching transcription factor binding sites in DNA sequences. Nucleic Acids Res. 2003;31: 3576-3579.

Podvinec M, Kaufmann MR, Handschin C, Meyer UA. NUBIScan, an in silico approach for prediction of nuclear receptor response elements. Mol Endocrinol 2002;16: 1269-1279.

Renisalo M, Putula J, Mannermaa E, Urtti A, Honkakoski P. Regulation of the human tyrosinase gene in retinal pigment epithelium cells: the significance of transcription factor orthodenticle homeobox 2 and its polymorphic binding site. Mol Vis. 2012;18: 38-54.

Sandelin A and Wasserman WW. Prediction of nuclear hormone receptor response elements. Mol Endocrinol 2005;19: 595-606.

Zancanella V, Giantin M, Lopparelli RM, Nebbia C, Dacasto M. Constitutive expression and phenobarbital modulation of drug metabolizing enzymes and related nuclear receptors in cattle liver and extra-hepatic tissues. Xenobiotica 2012;42: 1096-1109.
