# Supplementary material for: DNA elements for constitutive androstane receptor- and pregnane X receptor-mediated regulation of bovine CYP3A28 gene
Source: PLoS One. 2019 Mar 25;14(3):e0214338. doi: 10.1371/journal.pone.0214338 (PMC6433341; doi:10.1371/journal.pone.0214338)
Supplement: S1 Table — (PDF) [file pone.0214338.s002.pdf]

Title: DNA Elements for Constitutive Androstane Receptor- and Pregnane X Receptor-mediated Regulation of Bovine *CYP3A28* Gene

Authors: Mery Giantin, Jenni Küblbeck, Vanessa Zancanella, Viktoria Prantner, Fabiana Sansonetti, Axel Schoeniger, Roberta Tolosi, Giorgia Guerra, Silvia Da Ros, Mauro Dacasto, Paavo Honkakoski

Journal: Plos One

S1 Table. Primers used for the *ex novo* sequencing of bovine *CYP3A28* promoter region.

Amplified region	5'→3' sequences	Primer length (bp)	%GC	Tm (°C)	Amplicon size (bp)
-147	*F:* TGCATGTTATGCTCGGCCAACT	22	50.0	70.8	642
+495	*R:* ACACTGCACATGGGCCATTGTTT	23	47.8	71.5	
-1001	*F*: GCCCTTACCTCTACCAACTGAAT	23	47.8	63.6	1165
+164	*R*: ACTCACAGATAGAGGAGCACCAG	23	52.2	64.0	
-1567	*F*: TAATAAAATTTCCCTGTGCTCTGC	24	37.5	64.3	877
-690	*R*: ACCTAGTCTCTTCAGTGGATGGAG	24	50.0	64.1	
-1943	*F:* TGGTCCAGAGTACTGATAATGAGC	24	45.8	63.6	670
-1303	*R:* ACTTGTGGACACCTAGGGAATTT	23	43.4	63.9	
-2654	*F:* CTGAAGCTGCCATCCATTTCTC	22	50.0	67.3	795

-1859	*R:* ATCCACGTAACCCTGCTGAA	20	50.0	64.8	
-2598	*F:* CCAAAGACTCCAAAGGTATGC	22	47.6	62.8	611
-1987	*R:* TGTGAAAAACAAAACCTCAAAGG	23	34.7	64.2	
-3084	*F:* ACCCAGAGAAGGCACCAAAAGGGG	24	58.3	74.4	1229
-1855	*R:* AGGCATCCACGTAACCCTGCTGAA	24	54.1	73.3	
-3247	*F*: AGGAGTCCTTCAATCTCTGTCCAT	24	45.8	65.5	756
-2491	*R:* GCTGTGGGCTAGAAGCTATACAGT	24	50.0	64.0	
-3678	*F:* TGGGGAGTATAGACAGGTGACAAGCA	26	50.0	70.5	620
-3058	*R:* TGCCCCCTTTTGGTGCCTTCTCT	23	56.5	74.3	
-4184	*F:* GCTCTGACCATGCCAGGCCG	20	70.0	74.8	619
-3565	*R:* CTGCCCAGGGGCTCCTTCCA	20	70.0	75.0	
-4526	*F:* TCAGCCAGTGGAAGTCAGGACAGG	24	58.3	72.9	500
-4026	*R:* GGCATGGTCTTTGTGCCCTTCGTT	24	54.1	74.4	
-5053	*F:* TGAGATCTAGCTCCACATCCTA	22	45.4	60.9	664
-4389	*R:* GGCATTAGGCTCAGATATCAGGA	23	47.8	65.5	
-5369	*F:* TCCTCTGATTCCTCTTTCACTCC	23	47.8	65.0	443
-4926	*R:* CTATGTGAGGTAAGCAGTGTTGG	23	47.8	62.8	

-6347	*F:* AAACCCATAAGAGAAAGCACCTC	23	43.3	63.7	1096
-5251	*R:* CAGCAGATAAGGGATGTTGCAA	22	45.4	66.3	
-6830	*F:* ACCTTAGTAAGACCAGCAAGCA	22	45.4	62.2	679
-6151	*R:* CCATGTTGCCTTCTACTGTGG	21	52.3	64.8	
-7561	*F:* TCCTTGAAGAAGATTTGACTTCCAC	25	40.0	65.7	915
-6646	*R:* CAAACAGGTAGTCGCCACAG	20	55.0	63.8	
-7976	*F1:* TGAATTCGTGCAGTGAAGCC	20	50.0	66.7	665
-8581	*F2:* CATAGAGTGAGCTAGGCATGATTGAA	26	42.3	66.5	1270
-8605	*F3:* GGGTCAAGTTCATACTGTCCTCA	23	47.8	64.5	1294
-7311	*R:* GGGAATGTCAAAACTCACAAGCC	23	47.8	67.9	
-8471	*F:* CCCACTGGTGAGGCTGGGATAGG	23	65.2	73.6	625
-7846	*R:* TCCAGGGGAAGAGGGAAGAGGGT	23	60.8	72.9	
-8982	*F:* GTCATCTGTGGCAATCTTGATGC	23	47.8	68.1	694
-8288	*R:* TCGAGGATTCAGTTCCTCACTT	22	45.4	64.3	
-9656	*F:* CCTACTCAAACTCATGTCCATAGCAT	26	42.3	65.4	1248
-8408	*R:* GGGGGCACCCAATTAATATTCTAC	24	45.8	66.1	
-9456	*F:* TCCCTTTAGGATTGAATGGTTGG	23	43.4	66.9	593

-8863	*R:* GAAAGGAGTACGTCAAGGCTGT	22	50.0	63.8	
-10413	*F:* TAGAAAACTCTTGGTGCTGTATGC	24	41.6	63.6	874
-9539	*R:* CATTGGAAAAAGAAAGACTCTGGTC	25	40.0	65.2	
-10562	*F:* GTTGTGTCATAGTACCAGGCATA	23	43.4	60.8	376
-10186	*R:* TTTACCCTTTGAGAACTGACCAAC	24	41.6	64.4	

Positions of the amplified regions are relative to the transcription starting site (+1) of the bovine *CYP3A28* gene, coding sequence GenBank Acc. NM_001099367. The primer melting temperature (Tm) recommended for Phusion® High-Fidelity DNA Polymerase was calculated using a specific calculator freely available at http://www.thermoscientificbio.com/webtools/tmc/.
