# Supplementary material for: DNA elements for constitutive androstane receptor- and pregnane X receptor-mediated regulation of bovine CYP3A28 gene
Source: PLoS One. 2019 Mar 25;14(3):e0214338. doi: 10.1371/journal.pone.0214338 (PMC6433341; doi:10.1371/journal.pone.0214338)
Supplement: S2 Table — (PDF) [file pone.0214338.s003.pdf]

Title: DNA Elements for Constitutive Androstane Receptor- and Pregnane X Receptor-mediated Regulation of Bovine *CYP3A28* Gene

Authors: Mery Giantin, Jenni Küblbeck, Vanessa Zancanella, Viktoria Prantner, Fabiana Sansonetti, Axel Schoeniger, Roberta Tolosi, Giorgia Guerra, Silvia Da Ros, Mauro Dacasto, Paavo Honkakoski

Journal: Plos One

S2 Table. Oligonucleotide sequences for the amplification of the bovine *CYP3A28* promoter through long (ln) PCR reactions.

Start	5'→3' sequences	Length (bp)	%GC	Amplicon size (bp)	Reaction acronym
-2662	*F:* TGTTGGTACTGAAGCTGCCATC	22	50.0	2962	lnPCR#1
+387	*R:* GAATGTCACAAACCCTCCTAACAATG	26	42.3		
-7335	*F*: TGTGAGAACTGTGATCGTGCA	21	47.6	7635	lnPCR#2
+387	*R*: GAATGTCACAAACCCTCCTAACAATG	26	42.3		
-10651	*F*: TGTTGTGTCATAGTACCAGGCATA	24	41.7	8486	lnPCR#3
-2078	*R*: GTCTCACTTTCACTCCTTCTGCA	23	47.8		
-10651	*F:* TGTTGTGTCATAGTACCAGGCATA	24	41.7	3468	lnPCR#4
-7096	*R:* TCTTCTATCTGAAATGGGTCCAATCC	26	42.3		

Positions of the amplified regions are relative to the transcription starting site (+1) of the bovine *CYP3A28* gene, coding sequence GenBank ID NM_001099367.
