# Supplementary material for: DNA elements for constitutive androstane receptor- and pregnane X receptor-mediated regulation of bovine CYP3A28 gene
Source: PLoS One. 2019 Mar 25;14(3):e0214338. doi: 10.1371/journal.pone.0214338 (PMC6433341; doi:10.1371/journal.pone.0214338)
Supplement: S3 Table — (PDF) [file pone.0214338.s004.pdf]

Title: DNA Elements for Constitutive Androstane Receptor- and Pregnane X Receptor-mediated Regulation of Bovine *CYP3A28* Gene

Authors: Mery Giantin, Jenni Küblbeck, Vanessa Zancanella, Viktoria Prantner, Fabiana Sansonetti, Axel Schoeniger, Roberta Tolosi, Giorgia Guerra, Silvia Da Ros, Mauro Dacasto, Paavo Honkakoski

Journal: Plos One

S3 Table. Primer sequences used for nested PCR reactions of the bovine *CYP3A28* promoter.

Template	Start*	5'→3' sequences	Restriction enzymes	Length (bp)	%GC	Amplicon size (bp)	Product acronym
lnPCR#1	-284	*F:* CATAGATCTAGAGAGGTTTAGAGGAGA	BglII	27	40.7	355	PP
	+71	*R:* GATTCAAGCTTCTGAGTTTCCTTTCAG	HindIII	27	40.7		
lnPCR#1	-1359	*F*: GTTAGATCTCACTAGCAACACTTGG	BglII	25	44.0	1430	lnPP
	+71	*R*: GATTCAAGCTTCTGAGTTTCCTTTCAG	HindIII	27	40.7		
lnPCR#2	-3133	*F*: ATTGCTCGAGGATTGTTTACAGTTG	XhoI	25	40.0	2871	F1
	-262	*R*: CTCTGATCATCTCTGTCTCTATGGC	BclI	25	48.0		
lnPCR#3	-4998	*F:* CCCACTCGAGTTTTCTGTTTTTACA	XhoI	25	40.0	1865	F2
	-3133	*R:* GAGGATCCCATGGACAGAGATTGAA	BamHI	25	48.0		
lnPCR#3	-6899	*F*: CATCTCGAGTTACATTAGTTTCAGGC	XhoI	26	42.3	1962	F3
	-4937	*R*: AAGCAGTGTTGGGATTATTTGATTC	–	25	36.0		
lnPCR#3	-8314	*F:* CACAGCAAGTGAGGAACTGAATC	–	23	47.8	1562	F4

	-6752	*R:* ATGTTCACACAGGGAATAGAGTCAA	–	25	40.0		
lnPCR#4	-10368	*F:* GTGGTACCTGCACTAATGAGGAGGATT	KpnI	27	48.1	2115	F5
	-8259	*R:* GTCTGGATCCATTCAGATCGTCACAAG	BamHI	27	48.1		

Amplicons obtained from long PCR reactions (listed in Supplemental Table 2) were used as templates to amplify shorter promoter fragments. When needed, a selected restriction enzyme site was inserted in the primer sequence (underlined in the 5’→3’ sequence) for cloning in the parent plasmid pGL4.10[luc2] (proximal promoter, PP and long proximal promoter, lnPP) or the minimal promoter plasmid pGL4.10-PP (fragment F1-F5).
*: Positions relative to the transcription starting site (+1) of the bovine *CYP3A28* gene.
