# Supplementary material for: DNA elements for constitutive androstane receptor- and pregnane X receptor-mediated regulation of bovine CYP3A28 gene
Source: PLoS One. 2019 Mar 25;14(3):e0214338. doi: 10.1371/journal.pone.0214338 (PMC6433341; doi:10.1371/journal.pone.0214338)
Supplement: S4 Table — (PDF) [file pone.0214338.s005.pdf]

Title: DNA Elements for Constitutive Androstane Receptor- and Pregnane X Receptor-mediated Regulation of Bovine *CYP3A28* Gene

Authors: Mery Giantin, Jenni Küblbeck, Vanessa Zancanella, Viktoria Prantner, Fabiana Sansonetti, Axel Schoeniger, Roberta Tolosi, Giorgia Guerra, Silvia Da Ros, Mauro Dacasto, Paavo Honkakoski

Journal: Plos One

S4 Table. Oligonucleotides used for the internal site-directed mutagenesis of transcription factor binding sites.

Target motif	5'→3' sequences	Restriction enzyme site	Length (bp)	%GC
ER6	*F:* TTATTGATTTCCTACAGAATAactAgTCaAGGGAtGTaAAACTGAAGTGCATGTTAT	BcuI	57	31.6
	R: ATAACATGCACTTCAGTTTtACaTCCCTtGAcTagtTATTCTGTAGGAAATCAATAA		57	31.6
DR1	*F:* GGCTTTATTTTATTTTCTGTtACgccgcGGtTTACTGATAACACTGATTG	Cfr42I	50	38.0
	R: CAATCAGTGTTATCAGTAAaCCgcggcGTaACAGAAAATAAAATAAAGCC		50	38.0
DR5	*F:* AGGGAATTTCCTGGGTGAaaCtAGTCTAGcGTgAGATAAAACTCCTTAGAAGGTT	BcuI	55	41.8
	R: AACCTTCTAAGGAGTTTTATCTcACgCTAGACTaGttTCACCCAGGAAATTCCCT		55	41.8

Primers used for mutagenesis of ER6, DR1 present in the *CYP3A28* proximal promoter and DR5 located in F3 are indicated and lowercase letters refer to mutated bases. A selected restriction enzyme site was inserted in both primers (underlined in the 5'→3' sequence) for the diagnosis of successful base changing.
