# Supplementary material for: DNA elements for constitutive androstane receptor- and pregnane X receptor-mediated regulation of bovine CYP3A28 gene
Source: PLoS One. 2019 Mar 25;14(3):e0214338. doi: 10.1371/journal.pone.0214338 (PMC6433341; doi:10.1371/journal.pone.0214338)
Supplement: S5 Table — (PDF) [file pone.0214338.s006.pdf]

Title: DNA Elements for Constitutive Androstane Receptor- and Pregnane X Receptor-mediated Regulation of Bovine *CYP3A28* Gene

Authors: Mery Giantin, Jenni Küblbeck, Vanessa Zancanella, Viktoria Prantner, Fabiana Sansonetti, Axel Schoeniger, Roberta Tolosi, Giorgia Guerra, Silvia Da Ros, Mauro Dacasto, Paavo Honkakoski

Journal: Plos One

S5 Table. Oligonucleotides used in the inverse PCR procedure.

Template	5'→3' sequences	Length (bp)	%GC	Product acronym
PP	*sense:* AACTGAAGTGCATGTTATGCTCGCCAACTACCAAGGCAGAGAGG	44	50.0	PP_del
	antisense: GCCACTGGCTTTAATTCTCCTCTAAACCTCTCTAGATCTTGATATCCTCG	50	44.0	
PP+F3	*sense*: AACTGAAGTGCATGTTATGCTCGGCCAACTACCAAGGCAGAGAGG	45	51.1	PP_del+F3
	ansitsense: GCCACTGGCTTTAATTCTCCTCTAAACCTCTCTAGATCTCATAATTCT	48	39.6	
