# Supplementary material for: DNA elements for constitutive androstane receptor- and pregnane X receptor-mediated regulation of bovine CYP3A28 gene
Source: PLoS One. 2019 Mar 25;14(3):e0214338. doi: 10.1371/journal.pone.0214338 (PMC6433341; doi:10.1371/journal.pone.0214338)
Supplement: S6 Table — (PDF) [file pone.0214338.s007.pdf]

Title: DNA Elements for Constitutive Androstane Receptor- and Pregnane X Receptor-mediated Regulation of Bovine *CYP3A28* Gene

Authors: Mery Giantin, Jenni Küblbeck, Vanessa Zancanella, Viktoria Prantner, Fabiana Sansonetti, Axel Schoeniger, Roberta Tolosi, Giorgia Guerra, Silvia Da Ros, Mauro Dacasto, Paavo Honkakoski

Journal: Plos One

S6 Table. Oligonucleotides used in qPCR ChIP.

DNA region of interest	Primer sequence (5' – 3')	Length (bp)	% GC	Melting temperature (°C)
**ER6**	*F:* TGGACTACAGGCAGCCATAGA	21	52.4	60.3
	R: TGGTAGTTGGCCGAGCATAAC	21	52.4	60.4
**DR5**	*F:* TTTACCAGGCCCTGTCTCATG	21	52.4	59.7
	R: AGTGTTGGCCATCTCTCCTAATC	23	47.8	59.9
**EXON 13**	*F:* AAAGGTTGTGCTCAGAGATGGA	22	45.5	59.6
	R: AAGTAAATCAAGCCCCCTGAAATTC	25	40.0	59.8
