# Supplementary material for: DNA elements for constitutive androstane receptor- and pregnane X receptor-mediated regulation of bovine CYP3A28 gene
Source: PLoS One. 2019 Mar 25;14(3):e0214338. doi: 10.1371/journal.pone.0214338 (PMC6433341; doi:10.1371/journal.pone.0214338)
Supplement: S7 Table — (PDF) [file pone.0214338.s008.pdf]

Title: DNA Elements for Constitutive Androstane Receptor- and Pregnane X Receptor-mediated Regulation of Bovine *CYP3A28* Gene

Authors: Mery Giantin, Jenni Küblbeck, Vanessa Zancanella, Viktoria Prantner, Fabiana Sansonetti, Axel Schoeniger, Roberta Tolosi, Giorgia Guerra, Silvia Da Ros, Mauro Dacasto, Paavo Honkakoski

Journal: Plos One

S7 Table. bPXR-mediated transactivation of *CYP3A28* proximal promoter (PP) and fragment 3 (F3) using rifampicin (RIF).

		Mean luciferase activity* ± SD
**pGL4.10**	pCI-neo	100
	bPXR	140.53 ± 30.24
	bPXR+RIF	133.73 ± 52.57
**XREM**	pCI-neo	933.39 ± 509.69
	bPXR	1432.97 ± 842.79
	bPXR+RIF	2112.60 ± 972.68
**PP**	pCI-neo	3282.56 ± 3038.26
	bPXR	4618.43 ± 3841.19
	bPXR+RIF	4329.15 ± 3575.36
**PP+F3**	pCI-neo	26100.24 ± 21617.61
	bPXR	30173.12 ± 22813.48
	bPXR+RIF	32464.00 ± 24524.25

*C3A cells were transfected with the control reporter pCMVβ (150 ng/well), each reporter plasmids or CYP3A4-XREM-luc (XREM, 50 ng/well) and either bPXR expression plasmids or pCI-neo empty vector (25 ng/well). After transfection, cells were treated with vehicle (0.1% DMSO) or rifampicin (RIF, 10 μM) for 24 hours, and reporter activities were measured. Firefly luciferase activities were normalized with β-galactosidase activities. Data are expressed as mean luciferase activities ± SD (n = 3).
