# Supplementary material for: DNA elements for constitutive androstane receptor- and pregnane X receptor-mediated regulation of bovine CYP3A28 gene
Source: PLoS One. 2019 Mar 25;14(3):e0214338. doi: 10.1371/journal.pone.0214338 (PMC6433341; doi:10.1371/journal.pone.0214338)
Supplement: S8 Table — (PDF) [file pone.0214338.s009.pdf]

Title: DNA Elements for Constitutive Androstane Receptor- and Pregnane X Receptor-mediated Regulation of Bovine *CYP3A28* Gene

Authors: Mery Giantin, Jenni Küblbeck, Vanessa Zancanella, Viktoria Prantner, Fabiana Sansonetti, Axel Schoeniger, Roberta Tolosi, Giorgia Guerra, Silvia Da Ros, Mauro Dacasto, Paavo Honkakoski

Journal: Plos One

S8 Table. Modulation of *CYP2B22* mRNA in BFH12 cells exposed for 6 hours to different concentrations of phenobarbital (PB).

	CYP2B22* mRNA expression (AU, mean ± SD)
**DMSO 0.1%**	1.00
**PB 0.1 mM**	1.12 ± 0.27
**PB 0.25 mM**	0.98 ± 0.10
**PB 0.5 mM**	1.04 ± 0.19
**PB 1 mM**	1.06 ± 0.21
**PB 2 mM**	1.31 ± 0.04

*BFH12 cells were exposed to different concentrations (0.1, 0.25, 0.5, 1 and 2 mM) of phenobarbital (PB) for 6 hours. The expression of *CYP2B22* mRNA was detected by qPCR in control (0.1% DMSO) and treated cells, using *RPLP0* as internal control gene. The relative expression of DMSO-treated cells was set to 1 and its value was used for the normalization of the other groups. Data are expressed as the mean ± SD of two independent experiments (arbitrary units, AU). Statistical analysis: ANOVA + Tukey's post test.

*C3A cells were transfected with the control reporter pCMVβ (150 ng/well), each reporter plasmids or CYP3A4-XREM-luc (XREM, 50 ng/well) and either bPXR expression plasmids or pCI-neo empty vector (25 ng/well). After transfection, cells were treated with vehicle (0.1% DMSO) or rifampicin (RIF, 10 μM) for 24 hours, and reporter activities were measured. Firefly luciferase

activities were normalized with β-galactosidase activities. Data are expressed as mean luciferase activities ± SD (n = 3).
