# Supplementary material for: DNA elements for constitutive androstane receptor- and pregnane X receptor-mediated regulation of bovine CYP3A28 gene
Source: PLoS One. 2019 Mar 25;14(3):e0214338. doi: 10.1371/journal.pone.0214338 (PMC6433341; doi:10.1371/journal.pone.0214338)
Supplement: S1 Fig — Four CYP3A coding genes are known: CYP3A28, CYP3A38, CYP3A48 and the predicted CYP3A24. The GenBank IDs and nomenclature are displayed together with the nomenclature proposed by [27]. (PDF) [file pone.0214338.s010.pdf]

Title: DNA Elements for Constitutive Androstane Receptor- and Pregnane X Receptor-mediated Regulation of Bovine *CYP3A28* Gene

Authors: Mery Giantin, Jenni Küblbeck, Vanessa Zancanella, Viktoria Prantner, Fabiana Sansonetti, Axel Schoeniger, Roberta Tolosi, Giorgia Guerra, Silvia Da Ros, Mauro Dacasto, Paavo Honkakoski

Journal: Plos One

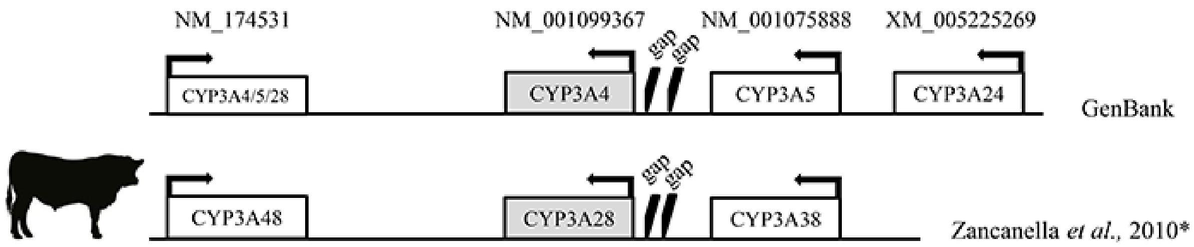

S1 Fig. Schematic organization of the bovine *CYP3A* locus. Four *CYP3A* coding genes are known: *CYP3A28*, *CYP3A38*, *CYP3A48* and the predicted *CYP3A24*. The GenBank IDs and nomenclature are displayed together with the nomenclature proposed by Zancanella et al. (2010).

*: Zancanella V, Giantin M, Lopparelli RM, Patarnello T, Dacasto M, Negrisolo E. Proposed new nomenclature for Bos taurus cytochromes P450 involved in xenobiotic drug metabolism. J Vet Pharmacol Ther. 2010; 33: 528-525.
