# Supplementary material for: DNA elements for constitutive androstane receptor- and pregnane X receptor-mediated regulation of bovine CYP3A28 gene
Source: PLoS One. 2019 Mar 25;14(3):e0214338. doi: 10.1371/journal.pone.0214338 (PMC6433341; doi:10.1371/journal.pone.0214338)
Supplement: S2 Fig — The re-sequencing of CYP3A28 promoter region was able to solve the first gap starting at –1492 bp revealing a sequencing artefact (A), as well as the second gap at base –2988 with a new genomic sequence (B). Two of the five single nucleotides polymorphisms (-2899T>G and -2981_-2979insA) are here shown circled. (PDF) [file pone.0214338.s011.pdf]

Title: DNA Elements for Constitutive Androstane Receptor- and Pregnane X Receptor-mediated Regulation of Bovine *CYP3A28* Gene

Authors: Mery Giantin, Jenni Küblbeck, Vanessa Zancanella, Viktoria Prantner, Fabiana Sansonetti, Axel Schoeniger, Roberta Tolosi, Giorgia Guerra, Silvia Da Ros, Mauro Dacasto, Paavo Honkakoski

Journal: Plos One

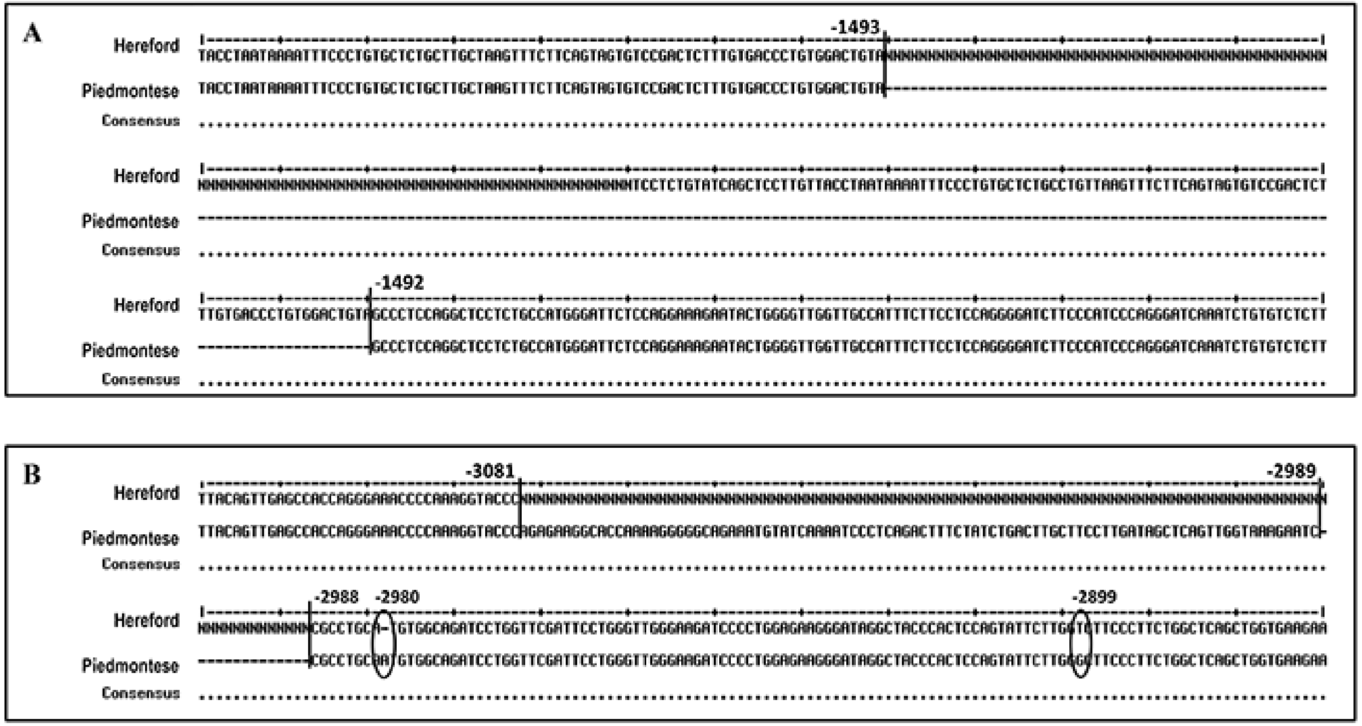

S2 Fig. Resolution of the two encountered gaps in the bovine *CYP3A28* promoter region. The re-sequencing of *CYP3A28* promoter region was able to solve the first gap starting at –1492 bp revealing a sequencing artefact (A), as well as the second gap at base –2988 with a new genomic sequence (B). Two of the five single nucleotides polymorphisms (-2899T>G and -2981_-2979insA) are here shown circled.
