# Supplementary material for: DNA elements for constitutive androstane receptor- and pregnane X receptor-mediated regulation of bovine CYP3A28 gene
Source: PLoS One. 2019 Mar 25;14(3):e0214338. doi: 10.1371/journal.pone.0214338 (PMC6433341; doi:10.1371/journal.pone.0214338)
Supplement: S3 Fig — Several constructs were produced to study the binding elements identified in the CYP3A28 proximal promoter (PP) and the contribution of the binding motif DR5 identified in F3. The parental PP was deleted of the whole putative region containing several TF binding-sites resulting in the PP_del; through site-direct direct mutagenesis the ER6 (PP_mER6) and DR1 (PP_mDR1) motifs were inactivated. The parental PP+F3 was deleted of the whole putative region containing several TF binding-sites resulting in the PP_del+F3; through site-direct direct mutagenesis the ER6 (PP_mER6+F3), the DR5 motif (PP+F3_mDR5 and PP_del+F3_mDR5) or both (PP_mER6+F3_mDR5) were inactivated. Details are reported in S1 File. Numbers indicate the positions relative to the transcriptional start site. C3A cells were transfected with the control reporter pCMVβ (150 ng/well), each reporter plasmid or PBREM-tk-luc (50 ng/well) and either bCAR expression plasmid or pCI-neo empty vector (25 ng/well). After transfection, cells were treated with vehicle (0.1% DMSO) for 24 hours, and reporter activities were measured. Firefly luciferase activities were normalized with β-galactosidase activities. Data are expressed as relative activities to those in pGL4.10 transfected cells (= 100) for each condition (pCI-neo empty or bCAR co-transfection). Data are the mean ± SD (n = 3 or 4). Results shown are representative of three independent assays. (PDF) [file pone.0214338.s012.pdf]

Title: DNA Elements for Constitutive Androstane Receptor- and Pregnane X Receptor-mediated Regulation of Bovine *CYP3A28* Gene

Authors: Mery Giantin, Jenni Küblbeck, Vanessa Zancanella, Viktoria Prantner, Fabiana Sansonetti, Axel Schoeniger, Roberta Tolosi, Giorgia Guerra, Silvia Da Ros, Mauro Dacasto, Paavo Honkakoski

Journal: Plos One

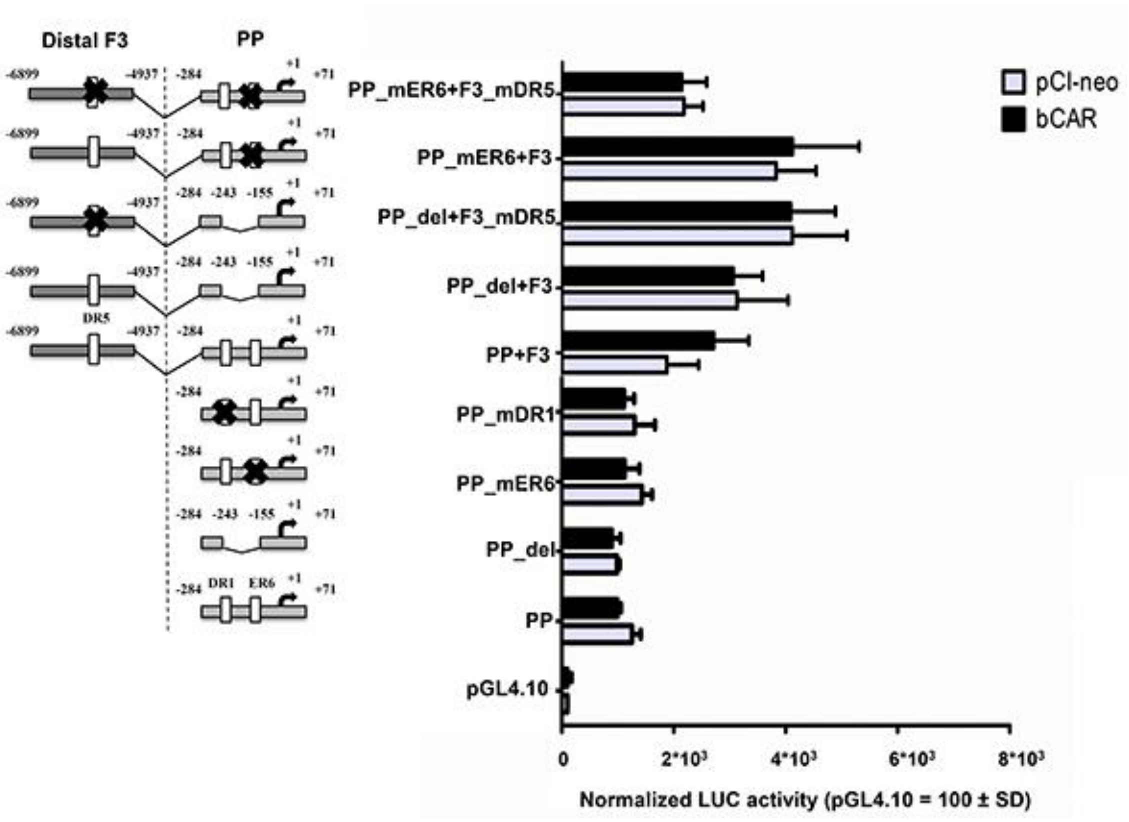

S3 Fig. Identification of bCAR-responsive elements in the proximal promoter and fragment 3 in *CYP3A28* promoter. Several constructs were produced to study the binding elements identified in the *CYP3A28* proximal promoter (PP) and the contribution of the binding motif DR5 identified in F3. The parental PP was deleted of the whole putative region containing several TF binding-sites resulting in the PP_del; through site-direct direct mutagenesis the ER6 (PP_mER6) and DR1 (PP_mDR1) motifs were inactivated. The parental PP+F3 was deleted of the whole putative region containing several TF binding-sites resulting in the PP_del+F3; through site-direct direct mutagenesis the ER6 (PP_mER6+F3), the DR5 motif (PP+F3_mDR5 and PP_del+F3_mDR5) or both (PP_mER6+F3_mDR5) were inactivated. Details are reported in S1 File. Numbers indicate the positions relative to the transcriptional start site. C3A cells were transfected with the control reporter pCMVβ (150 ng/well), each reporter plasmid or PBREM-tk-luc (50 ng/well) and either bCAR expression plasmid or pCI-neo empty vector (25 ng/well). After transfection, cells were treated with vehicle (0.1% DMSO) for 24 hours, and reporter activities were measured. Firefly luciferase activities were normalized with β-galactosidase activities. Data are expressed as relative activities to those in pGL4.10 transfected cells (= 100) for each condition (pCI-neo empty or bCAR co-transfection). Data are the mean ± SD (n = 3 or 4). Results shown are representative of three independent assays.
