# Supplementary material for: DNA elements for constitutive androstane receptor- and pregnane X receptor-mediated regulation of bovine CYP3A28 gene
Source: PLoS One. 2019 Mar 25;14(3):e0214338. doi: 10.1371/journal.pone.0214338 (PMC6433341; doi:10.1371/journal.pone.0214338)
Supplement: S5 Fig — BFH12 cells were treated with different CYP3A inducers (PCN, RU486, SR12813, DEX and RIF) at the fixed concentration 10 μM for 0, 1, 3, 6, 12 and 24 hours. The expression of CAR (A), PXR (B) and RXRα (C) was detected by qPCR in control (0.1% DMSO) and treated cells, using RPLP0 as internal control gene. The relative expression of DMSO-treated cells was set to 1 and its value was used for the normalization of the other groups. Data are expressed as the mean ± SD of three independent experiments (arbitrary units, AU). Statistical analysis: ANOVA + Tukey’s post test. (PDF) [file pone.0214338.s014.pdf]

Title: DNA Elements for Constitutive Androstane Receptor- and Pregnane X Receptor-mediated Regulation of Bovine *CYP3A28* Gene

Authors: Mery Giantin, Jenni Küblbeck, Vanessa Zancanella, Viktoria Prantner, Fabiana Sansonetti, Axel Schoeniger, Roberta Tolosi, Giorgia Guerra, Silvia Da Ros, Mauro Dacasto, Paavo Honkakoski

Journal: Plos One

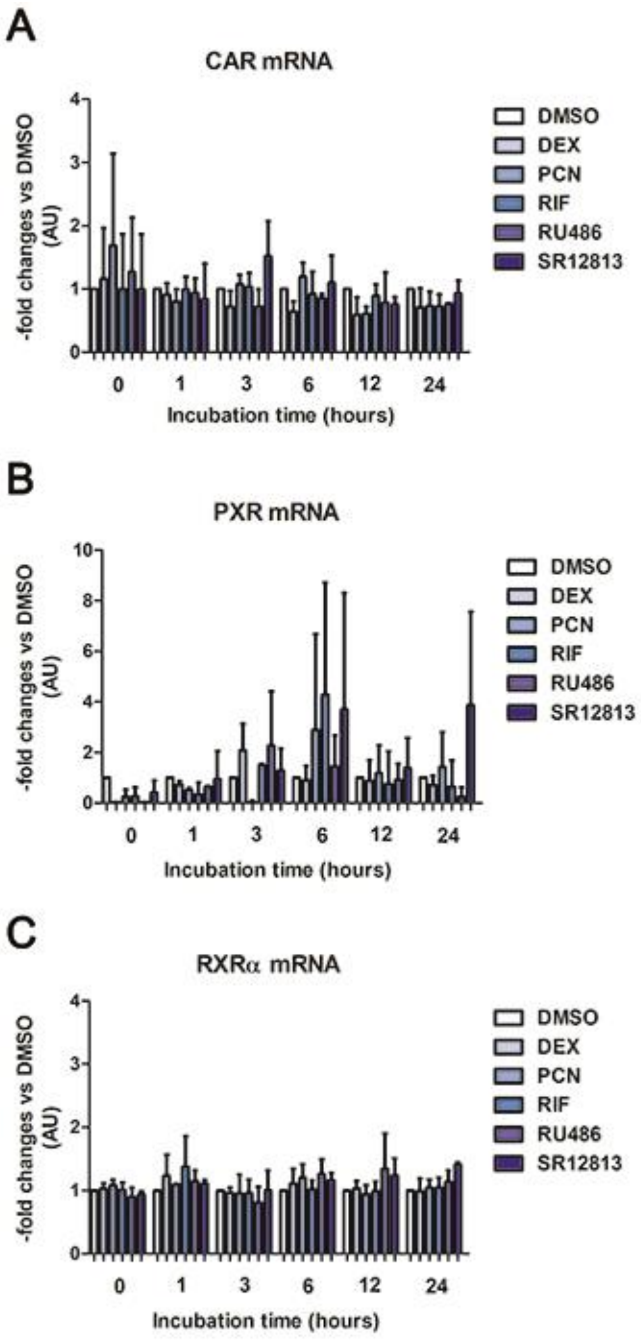

S5 Fig. Induction of *CAR*, *PXR*, *RXRα* mRNAs in BFH12 cells exposed for 0, 1, 3, 6, 12 and 24 hours to five prototypical CYP3A inducers. BFH12 cells were treated with different CYP3A inducers (PCN, RU486, SR12813, DEX and RIF) at the fixed concentration 10 µM for 0, 1, 3, 6, 12 and 24 hours. The expression of *CAR* (A), *PXR* (B) and *RXRα* (C) was detected by qPCR in control (0.1% DMSO) and treated cells, using *RPLP0* as internal control gene. The relative expression of DMSO-treated cells was set to 1 and its value was used for the normalization of the other groups. Data are expressed as the mean ± SD of three independent experiments (arbitrary units, AU). Statistical analysis: ANOVA + Tukey's post test.
