# Supplementary material for: DNA elements for constitutive androstane receptor- and pregnane X receptor-mediated regulation of bovine CYP3A28 gene
Source: PLoS One. 2019 Mar 25;14(3):e0214338. doi: 10.1371/journal.pone.0214338 (PMC6433341; doi:10.1371/journal.pone.0214338)
Supplement: S6 Fig — BFH12 cells were treated with different concentrations of SR12813 (1, 2.5, 5, 10, 25 μM) and RIF (1, 2.5, 5, 10, 25, 50 and 100 μM) for 6 hours, as described in Materials and Methods. The expression of CYP3A28 was detected by qPCR in control (0.1% DMSO) and treated cells, using RPLP0 as internal control gene. The relative expression of DMSO-treated cells was set to 1 and its value was used for the normalization of the other groups. Data are expressed as the mean ± SD of two independent experiments (arbitrary units, AU). Statistical analysis: ANOVA + Tukey’s post-test. (PDF) [file pone.0214338.s015.pdf]

Title: DNA Elements for Constitutive Androstane Receptor- and Pregnane X Receptor-mediated Regulation of Bovine *CYP3A28* Gene

Authors: Mery Giantin, Jenni Küblbeck, Vanessa Zancanella, Viktoria Prantner, Fabiana Sansonetti, Axel Schoeniger, Roberta Tolosi, Giorgia Guerra, Silvia Da Ros, Mauro Dacasto, Paavo Honkakoski

Journal: Plos One

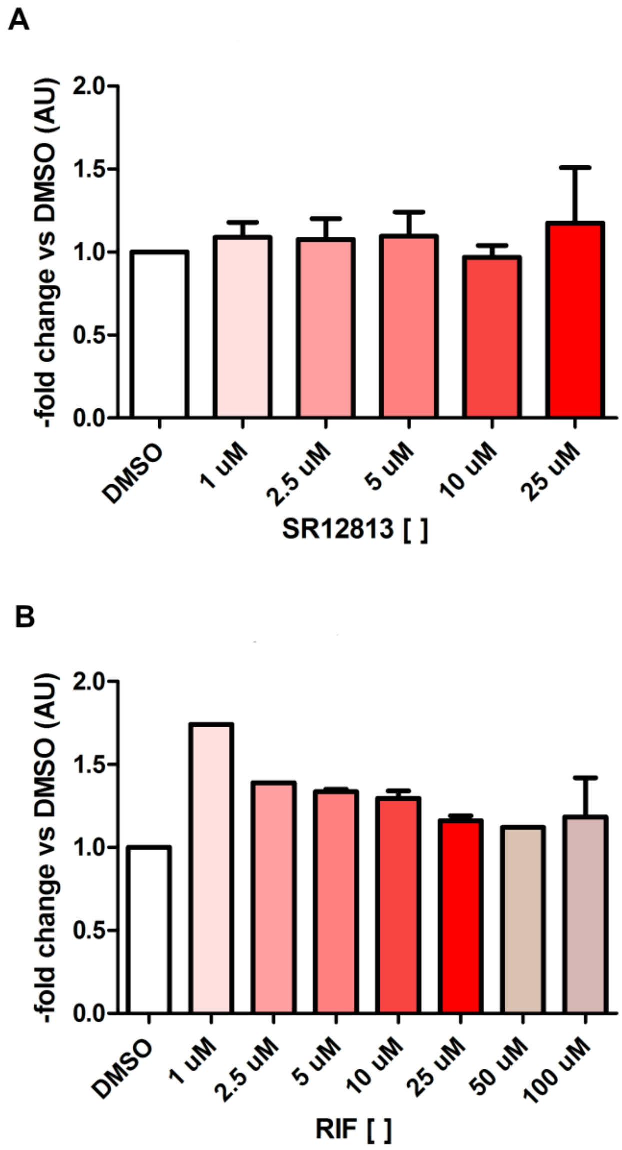

S6 Fig. Induction of *CYP3A28* mRNA in BFH12 cells exposed to increasing concentrations of SR12813 and RIF for 6 hours. BFH12 cells were treated with different concentrations of SR12813 (1, 2.5, 5, 10, 25 µM) and RIF (1, 2.5, 5, 10, 25, 50 and 100 µM) for 6 hours, as described in Materials and Methods. The expression of *CYP3A28* was detected by qPCR in control (0.1% DMSO) and treated cells, using *RPLP0* as internal control gene. The relative expression of DMSO-treated cells was set to 1 and its value was used for the normalization of the other groups. Data are expressed as the mean ± SD of two independent experiments (arbitrary units, AU). Statistical analysis: ANOVA + Tukey's post test.
