# Supplementary material for: DNA elements for constitutive androstane receptor- and pregnane X receptor-mediated regulation of bovine CYP3A28 gene
Source: PLoS One. 2019 Mar 25;14(3):e0214338. doi: 10.1371/journal.pone.0214338 (PMC6433341; doi:10.1371/journal.pone.0214338)
Supplement: S7 Fig — BFH12 cells were exposed to different concentrations (1, 3, 10 and 30 μM) of the bCAR activator, FL81, for 6 and 12 hours. The expression of CYP3A28 (A, B) and CYP2B22 (C, D) mRNA was detected by qPCR in control (0.1% DMSO) and treated cells, using RPLP0 as internal control gene. The relative expression of DMSO-treated cells was set to 1 and its value was used for the normalization of the other groups. Data are expressed as the mean ± SD of three independent experiments (arbitrary units, AU). Statistical analysis: ANOVA + Tukey’s post-test. Significance was defined as P < 0.01: **; P < 0.001: ***. A, C: 6 hours of incubation; B, D: 12 hours of incubation. (PDF) [file pone.0214338.s016.pdf]

Title: DNA Elements for Constitutive Androstane Receptor- and Pregnane X Receptor-mediated Regulation of Bovine *CYP3A28* Gene

Authors: Mery Giantin, Jenni Küblbeck, Vanessa Zancanella, Viktoria Prantner, Fabiana Sansonetti, Axel Schoeniger, Roberta Tolosi, Giorgia Guerra, Silvia Da Ros, Mauro Dacasto, Paavo Honkakoski

Journal: Plos One

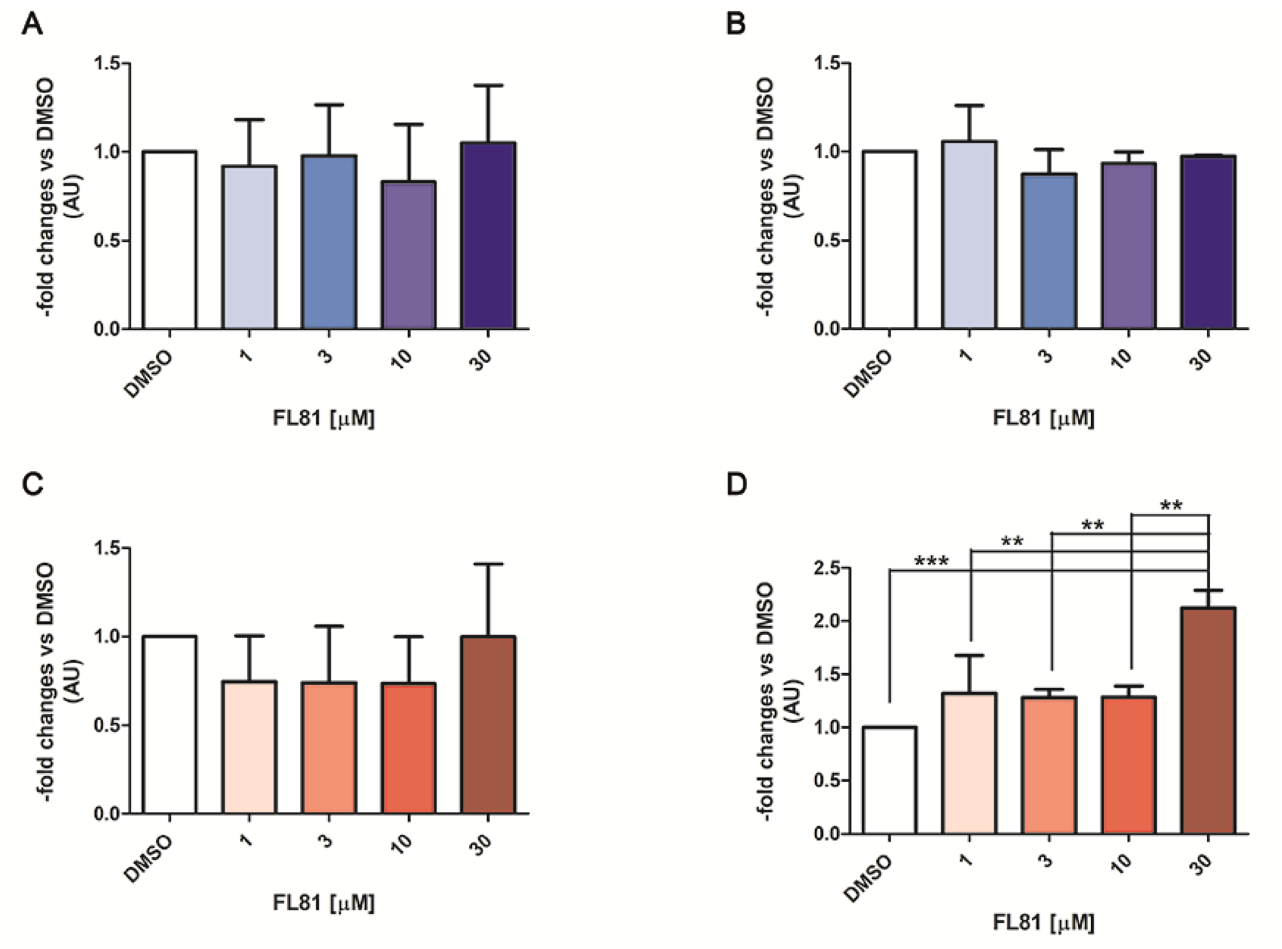

S7 Fig. Induction of *CYP3A28* and *CYP2B22* mRNA in BFH12 cells exposed for 6 and 12 hours to FL81. BFH12 cells were exposed to different concentrations (1, 3, 10 and 30 µM) of the bCAR activator, FL81, for 6 and 12 hours. The expression of *CYP3A28* (A, B) and *CYP2B22* (C, D) mRNA was detected by qPCR in control (0.1% DMSO) and treated cells, using *RPLP0* as internal control gene. The relative expression of DMSO-treated cells was set to 1 and its value was used for the normalization of the other groups. Data are expressed as the mean ± SD of three independent experiments (arbitrary units, AU). Statistical analysis: ANOVA + Tukey's post test. Significance was defined as $P < 0.01$: **; $P < 0.001$: ***. A, C: 6 hours of incubation; B, D: 12 hours of incubation.
