# Supplementary material for: DNA elements for constitutive androstane receptor- and pregnane X receptor-mediated regulation of bovine CYP3A28 gene
Source: PLoS One. 2019 Mar 25;14(3):e0214338. doi: 10.1371/journal.pone.0214338 (PMC6433341; doi:10.1371/journal.pone.0214338)
Supplement: S8 Fig — Bovine liver cytosolic and nuclear extracts were isolated according to Renisalo et al. (2012) with minor modifications. Proteins (30 μg) were subjected to immunoblotting analysis following the protocol previously published by [14]. Membranes were firstly probed with anti-human CAR (1:1000 final dilution), anti-human PXR (1:1000) and anti-human RXR (1:1500) polyclonal antibodies and then with a peroxidase-conjugated goat anti-rabbit IgG (Chemicon International; 1:6000 final dilution). As positive control, total proteins isolated from C3A cells stably transfected with hCAR or hPXR [41] was used. 1: molecular weight marker (ChemiBlot Molecular Weight Marker, Millipore); 2: C3A cells transfected with hPXR or hCAR, total proteins; 3: bovine liver tissue, cytosol; 4: bovine liver tissue, nuclear fraction. (PDF) [file pone.0214338.s017.pdf]

Title: DNA Elements for Constitutive Androstane Receptor- and Pregnane X Receptor-mediated Regulation of Bovine *CYP3A28* Gene

Authors: Mery Giantin, Jenni Küblbeck, Vanessa Zancanella, Viktoria Prantner, Fabiana Sansonetti, Axel Schoeniger, Roberta Tolosi, Giorgia Guerra, Silvia Da Ros, Mauro Dacasto, Paavo Honkakoski

Journal: Plos One

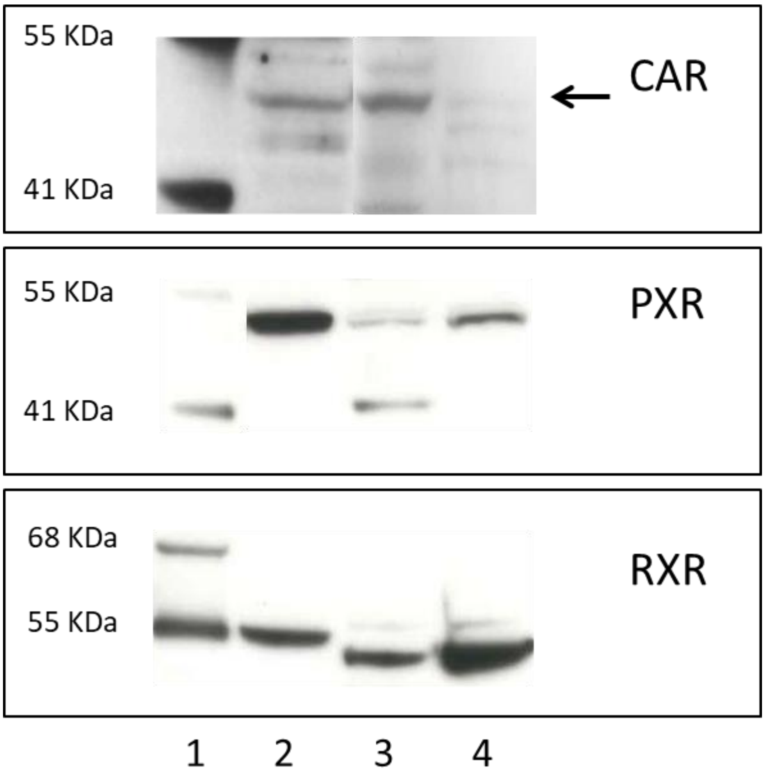

S8 Fig. CAR, PXR and RXR immunoblotting analysis of subcellular fractions isolated from untreated bovine liver. Bovine liver cytosolic and nuclear extracts were isolated according to Renisalo et al. (2012) with minor modifications. Proteins (30 µg) were subjected to immunoblotting analysis following the protocol previously published by Zancanella et al. (2012)*. Membranes were firstly probed with anti-human CAR (1:1000 final dilution), anti-human PXR (1:1000) and anti-human RXR (1:1500) polyclonal antibodies and then with a peroxidase-conjugated goat anti-rabbit IgG (Chemicon International; 1:6000 final dilution). As positive control, total proteins isolated from C3A cells stably transfected with hCAR or hPXR (Küblbeck et al., 2010) was used. 1: molecular weight marker (ChemiBlot Molecular Weight Marker, Millipore); 2: C3A cells transfected with hPXR or hCAR, total proteins; 3: bovine liver tissue, cytosol; 4: bovine liver tissue, nuclear fraction.

* Zancanella V, Giantin M, Lopparelli RM, Nebbia C, Dacasto M. Constitutive expression and phenobarbital modulation of drug metabolizing enzymes and related nuclear receptors in cattle liver and extra-hepatic tissues. Xenobiotica 2012; 42: 1096-1109.
