# Supplementary material for: DNA elements for constitutive androstane receptor- and pregnane X receptor-mediated regulation of bovine CYP3A28 gene
Source: PLoS One. 2019 Mar 25;14(3):e0214338. doi: 10.1371/journal.pone.0214338 (PMC6433341; doi:10.1371/journal.pone.0214338)
Supplement: S10 Fig — BFH12 cells were exposed to 0.1% DMSO for 6 hours. Chromatin was then isolated, subjected to ChIP using anti-human CAR antibody and quantified by qPCR as described in S1 File. Results for both ER6 and DR5 DNA regions are reported. The data shown derived from two further independent experiments (panels A, B); they are normalized to input DNA and expressed as % ChIP/input. The experiment was performed four times independently, and similar results were obtained. Chromatin samples from control cells immunoprecipitated with or without Histone H3 antibody are shown as Histone H3 and beads, respectively. A further negative control (exon 13), representing a CYP3A28 DNA region without NR binding sites, is reported in the graph. In all experiments, negative and positive controls behaved as expected. (PDF) [file pone.0214338.s019.pdf]

Title: DNA Elements for Constitutive Androstane Receptor- and Pregnane X Receptor-mediated Regulation of Bovine *CYP3A28* Gene

Authors: Mery Giantin, Jenni Küblbeck, Vanessa Zancanella, Viktoria Prantner, Fabiana Sansonetti, Axel Schoeniger, Roberta Tolosi, Giorgia Guerra, Silvia Da Ros, Mauro Dacasto, Paavo Honkakoski

Journal: Plos One

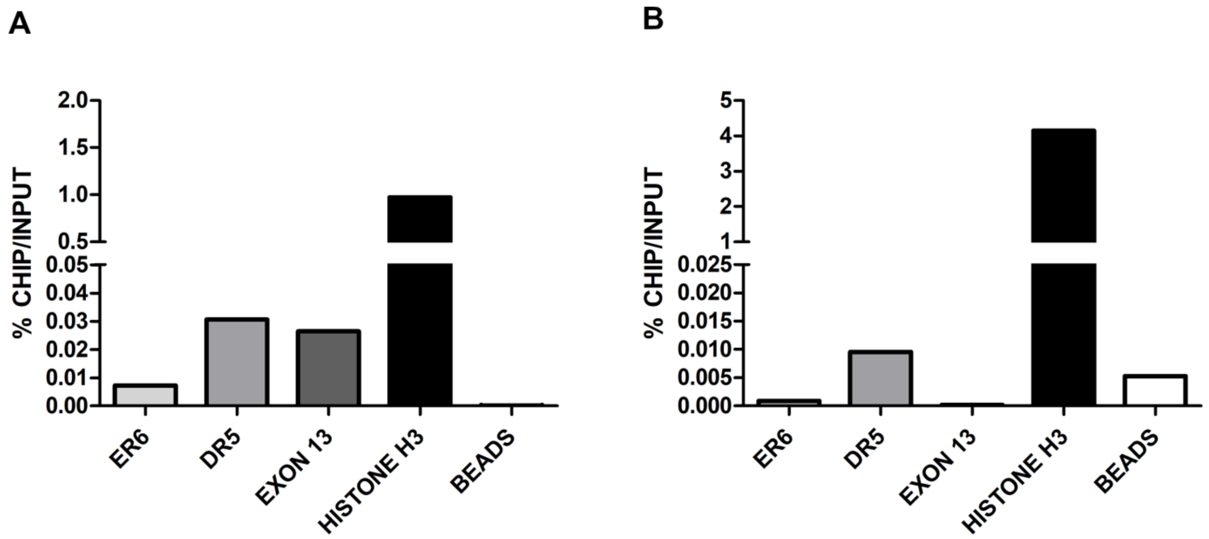

S10 Fig. ChIP in control BFH12 cells to quantify the binding of CAR to ER6 and DR5 binding sites. BFH12 cells were exposed to 0.1% DMSO for 6 hours. Chromatin was then isolated, subjected to ChIP using anti-human CAR antibody and quantified by qPCR as described in S1 File. Results for both ER6 and DR5 DNA regions are reported. The data shown derived from two further independent experiments (panels A, B); they are normalized to input DNA and expressed as % ChIP/input. The experiment was performed four times independently, and similar results were obtained. Chromatin samples from control cells immunoprecipitated with or without Histone H3 antibody are shown as Histone H3 and beads, respectively. A further negative control (exon 13), representing a *CYP3A28* DNA region without NR binding sites, is reported in the graph. In all experiments, negative and positive controls behaved as expected.
