# Supplementary material for: DNA elements for constitutive androstane receptor- and pregnane X receptor-mediated regulation of bovine CYP3A28 gene
Source: PLoS One. 2019 Mar 25;14(3):e0214338. doi: 10.1371/journal.pone.0214338 (PMC6433341; doi:10.1371/journal.pone.0214338)
Supplement: S11 Fig — BFH12 cells were exposed to 0.1% DMSO and 100 μM RU486 for 6 hours. Chromatin was isolated, subjected to ChIP using anti-human PXR antibody and quantified by qPCR as described in Material and Methods. Results for ER6 and DR5 DNA regions are reported in panel A and B, respectively. Data are normalized to input DNA and expressed as % ChIP/input. The experiment was performed four times independently, and similar results were obtained. The data shown derived from a representative experiment. Chromatin samples from control cells immunoprecipitated with or without Histone H3 antibody are shown as Histone H3 and beads, respectively. A further negative control (exon 13), representing a CYP3A28 DNA region without NR binding sites, is reported in the graph. In all experiments, negative and positive controls behave as expected. (PDF) [file pone.0214338.s020.pdf]

Title: DNA Elements for Constitutive Androstane Receptor- and Pregnane X Receptor-mediated Regulation of Bovine *CYP3A28* Gene

Authors: Mery Giantin, Jenni Küblbeck, Vanessa Zancanella, Viktoria Prantner, Fabiana Sansonetti, Axel Schoeniger, Roberta Tolosi, Giorgia Guerra, Silvia Da Ros, Mauro Dacasto, Paavo Honkakoski

Journal: Plos One

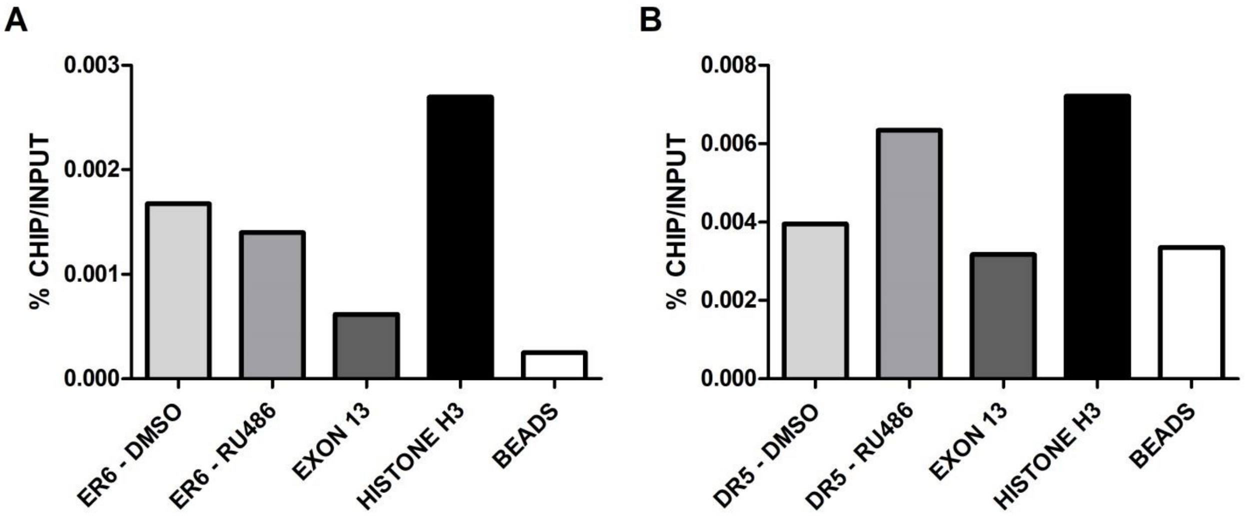

S11 Fig. ChIP in control and treated BFH12 cells to quantify the binding of PXR to ER6 and DR5 binding sites. BFH12 cells were exposed to 0.1% DMSO and 100 µM RU486 for 6 hours. Chromatin was isolated, subjected to ChIP using anti-human PXR antibody and quantified by qPCR as described in Material and Methods. Results for ER6 and DR5 DNA regions are reported in panel A and B, respectively. Data are normalized to input DNA and expressed as % ChIP/input. The experiment was performed four times independently, and similar results were obtained. The data shown derived from a representative experiment. Chromatin samples from control cells immunoprecipitated with or without Histone H3 antibody are shown as Histone H3 and beads, respectively. A further negative control (exon 13), representing a *CYP3A28* DNA region without NR binding sites, is reported in the graph. In all experiments, negative and positive controls behave as expected.
